# YAP Dictates Mitochondrial Redox Homeostasis to Facilitate Obesity‐Associated Breast Cancer Progression

**DOI:** 10.1002/advs.202103687

**Published:** 2022-02-18

**Authors:** Jia‐Zih Dai, Yen‐Ju Wang, Cheng‐Hsun Chen, I‐Lin Tsai, Yi‐Chun Chao, Cheng‐Wei Lin

**Affiliations:** ^1^ Department of Biochemistry and Molecular Cell Biology School of Medicine College of Medicine Taipei Medical University Taipei 110 Taiwan; ^2^ Graduate Institute of Medical Sciences College of Medicine Taipei Medical University Taipei 110 Taiwan; ^3^ Cell Physiology and Molecular Image Research Center Wan Fang Hospital Taipei Medical University Taipei 116 Taiwan; ^4^ Drug Development and Value Creation Research Center Kaohsiung Medical University Kaohsiung 807 Taiwan

**Keywords:** breast cancer, fatty acid oxidation, obesity, oxidative phosphorylation, YAP

## Abstract

Dysregulation of hormones is considered a risk factor for obesity‐mediated breast tumorigenesis; however, obesity is associated with poor outcomes among women diagnosed with triple‐negative breast cancer (TNBC), which is a hormone‐independent breast cancer subtype. Thus, identifying the driving force behind the obesity‐breast cancer relationship is an urgent need. Here it is identified that diet‐induced obesity (DIO) facilitates tumorigenesis of TNBC cells. Mechanistically, DIO induces a metabolic addiction to fatty acid oxidation (FAO), accompanied by coordinated activation of Yes‐associated protein (YAP) signaling. Specifically, YAP governs mitochondrial redox homeostasis via transcriptional regulation of antioxidant‐related enzymes, which renders tumor cells capable of extenuating FAO‐elicited mitochondrial oxidative stress. Moreover, adipocytes‐derived fatty acids are identified to be responsible for enhancing the FAO‐YAP axis and antioxidative capacity, and higher expression of an obesity signature in breast cancer patients is positively correlated with YAP signaling and antioxidant genes. The findings uncover the crucial role of YAP in dictating mitochondrial redox homeostasis for obesity‐mediated metabolic adaptation and breast tumor progression.

## Graphical abstract

It is identified that Yes‐associated protein (YAP) governs mitochondria redox homeostasis through the regulation of antioxidant gene expressions in obesity‐associated breast cancer cells. Activation of YAP alleviates metabolic stress from fatty acid oxidation, resulting in decreased metabolic stress and the promotion of a survival advantage.

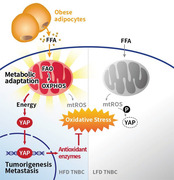

## 1 Introduction

Breast cancer is the most often diagnosed cancer and the second leading cause of cancer‐related deaths in women. Two million new cases are reported and half a million deaths occur worldwide every year.^[^ ^1^ ^]^ Women with metabolic disorders, including obesity and diabetes, have an increased risk of developing breast cancer.^[^ ^2^ ^]^ Obese cancer patients were reported to be associated with an increased risk of recurrence, metastasis, and poor response to first‐line therapies and exhibited poorer survival outcomes.^[^ ^3^ ^]^ Although dysregulation of hormones is considered a driving force in breast tumorigenesis,^[^ ^4^ ^]^ obesity is strongly associated with poor outcomes among women diagnosed with triple‐negative breast cancer (TNBC),^[^ ^5^ ^]^ which is hormone independent and the most aggressive breast cancer subtype. These results imply that the underlying regulatory mechanism is far more complicated and urgently needs to be elucidated.

Reprogramming of metabolic pathways enables tumor cells to rapidly proliferate and survive in conditions of nutrient depletion and hypoxia, and to evade immune surveillance.^[^ ^6^ ^]^ Aerobic glycolysis, also known as the Warburg effect, is a well‐documented metabolic symbol of cancer, as it describes how tumor cells rely on glycolysis to generate energy even in an aerobic environment. In addition to aerobic glycolysis, tumor cells adopt different metabolic pathways such as fatty acid oxidation (FAO) and glutaminolysis in different situations.^[^ ^7^ ^]^ Studies have reported that breast and colorectal tumor cells have a predilection to spread to adipocyte‐rich tissues.^[^ ^8^ ^]^ Adipocyte‐derived factor, such as leptin, upregulated FAO activity to support breast cancer stemness properties.^[^ ^9^ ^]^ Additionally, obesity induces global metabolic changes in both tumors and cells in the tumor microenvironment (TME).^[^ ^10^ ^]^ It was also found that adipocytes predispose tumor cells and tumor‐infiltrating myeloid‐derived suppressor cells (MDSCs) to increased fatty acid uptake and FAO activity.^[^ ^11^ ^]^ Obesity remodels fatty acid utilization in both tumor cells and the TME to suppress antitumor immunity^[^ ^10^ ^]^ and induces FAO in cluster of differentiation 8‐positive (CD8+) effector T cells, which is crucial for obesity‐promoted breast tumor growth.^[^ ^12^ ^]^ These findings suggest that targeting cancer metabolism could be a promising therapeutic approach in both tumors and the TME. However, the contribution of obesity‐mediated metabolic reprogramming to breast cancer development and the detailed mechanism linking adipocytes and metabolic switching in breast tumor cells remain unknown.

In the present study, we provide a fundamental understanding of the role of metabolic alterations in obesity‐associated breast cancer progression. Using a diet‐induced obesity (DIO) syngeneic mouse model and multiomics approaches, we found that obesity induced metabolic reprogramming to FAO and mitochondrial oxidative phosphorylation (OXPHOS) which was accompanied by coordinated activation of Yes‐associated protein (YAP) signaling. Notably, activation of YAP induced expressions of antioxidant genes to alleviate increased mitochondrial reactive oxygen species (ROS) levels and oxidative stress due to lipid oxidation. In addition, obesity‐associated adipocytes play a role in regulating YAP signaling and the antioxidative capacity of TNBC cells. Our findings showed that YAP acts as a master regulator of mitochondrial redox homeostasis to adapt to lipid metabolism‐elicited oxidative stress. This work sheds light on how metabolic adaptation via mitochondrial redox homeostasis is related to obesity‐associated breast cancer progression.

## 2 Results

### 2.1 Obesity Promotes Breast Cancer Progression and Induces a Global Metabolic Switch

**Figure 1:**
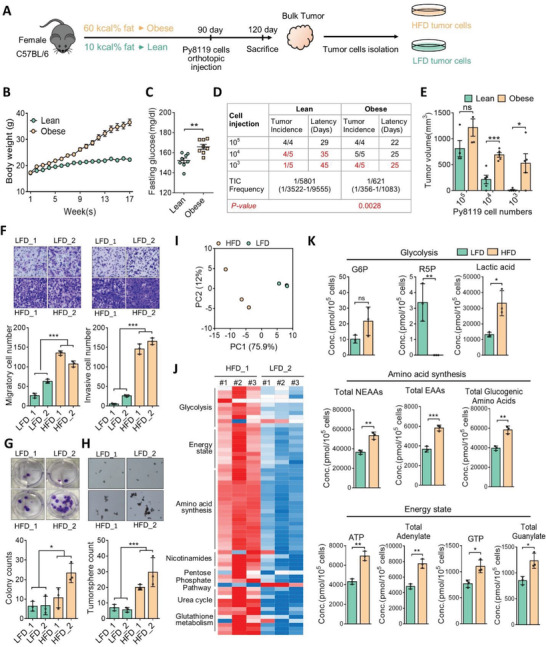
DIO promotes TNBC progression and alters tumor metabolism. A) Female C57BL/6 mice were fed diets with 10 kcal%/no sucrose (lean) or 60 kcal% fat (DIO) for 12 weeks and B) body weights (*n *= 14/group) and C) fasting glucose concentrations (*n *= 8/group) were measured. D) Limiting dilution assay of Py8199 cells injected orthotopically into the mammary fat pad of DIO and lean C57BL/6 mice (*n *= 4 or 5/group). Tumor‐initiating cell (TIC) frequency was calculated by l‐Calc software. E) Tumor weights (*n *= 4 or 5/group) were measured at the end of the experiment. F–H) Ex vivo analysis of tumor‐migration/invasion, mammosphere‐formation, and colony‐formation capabilities in high‐fat diet (HFD) and low‐fat diet (LFD) tumor cells respectively isolated from DIO and lean mice. Representative images of migration, invasion, colony, and mammosphere formation (upper panel), and quantitative data (lower panel) are shown. I–K) Metabolome analysis of HFD and LFD cells. I) Principal component analysis plot of metabolomics data. J) Heatmap of relative levels of metabolites in HFD or LFD cells. Data were derived from three biological repeats. K) Absolute quantitative analysis of metabolites in HFD or LFD cells. The metabolic parameters for glycolysis, amino acid synthesis, and energy status are shown. Data are expressed as the mean ± standard deviation (SD) of at least three replicates. * *p* < 0.05, ** *p* < 0.01, *** *p* < 0.001, as determined by an unpaired two‐tailed Student's *t*‐test.

To evaluate the effect of obesity on breast tumor progression, a DIO model was created in C57BL/6 mice fed a diet containing 60 kcal% fat or 10 kcal% fat for 12 weeks (**Figure** **1**A). The body weight and fasting glucose concentration had significantly increased in obese mice after 8 weeks, compared to the lean group (Figure 1B,C). Then, mouse TNBC Py8119 cells were orthotopically implanted into the mammary fat pad of mice in serially diluted cell numbers and monitored for another 4 weeks (Figure 1A). Results showed that DIO significantly promoted the tumor‐initiating capacity (TIC) and growth of Py8119 cells (Figure 1D,E). TIC frequencies in obese and lean mice were 1/621 and 1/5801, respectively (Figure 1D). The primary tumor tissues from four individual mice were subsequently isolated from obese and lean mice and defined as high‐fat diet (HFD#1 and HFD#2) and low‐fat diet (LFD#1 and LFD#2) tumor cells, respectively (Figure 1A). Ex vivo functional studies identified that aggressive features of the tumors, including invasion, proliferation, and tumorsphere formation, were substantially upregulated in HFD cells, compared to LFD cells (Figure 1F–H). To explore the involvement of metabolic switching in obesity‐associated tumor progression, metabolomics analyses were further applied. Heat map and principle component analyses illustrated global changes in central carbon metabolites involved in glycolysis, amino acid biosynthesis, and energy production in HFD cells (Figure 1I–K and Figure S1, Supporting Information). These data suggested that obesity‐driven breast tumor progression may be associated with alterations in cellular metabolism.

### 2.2 Obesity‐Associated Breast Cancer Cells Are Addicted to Fatty Acid Oxidation

**Figure 2:**
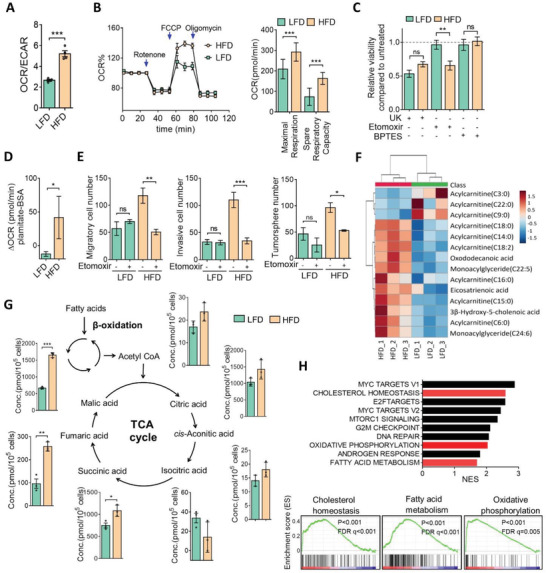
Obesity‐associated breast cancer cells are addicted to FAO. A) The basal oxygen consumption rate (OCR) to extra cellular acidification rate (ECAR) ratio in HFD or LFD cells. B) The OCR was measured for HFD or LFD cells with a Seahorse XFe24 Flux Analyzer. The maximal respiration and spare respiration capacities (right panel) are shown. C) HFD or LFD cells were treated with UK5099 (20 × 10^−6^ m), etomoxir (100 × 10^−6^ m), or Bis‐2‐(5‐phenylacetamido‐1,3,4‐thiadiazol‐2‐yl)ethyl sulfide (BPTES (40 × 10^−6^ m) for 72 h, and cell viability was measured by a CCK8 assay. D) FAO dependency in HFD and LFD cells. The Δ oxygen consumption rate (OCR) was calculated as the OCR at the time of the BSA‐conjugated palmitate injection. E) HFD or LFD cells were treated with etomoxir (100 × 10^−6^ m) for 24 h and the migration, invasion, and mammosphere‐formation abilities were assessed. F) Heatmap showing relative FAO‐associated metabolites in LFD and HFD cells, as analyzed by ultra performance liquid chromatography (UPLC). G) Absolute quantitative analysis of tricarboxylic acid (TCA) cycle‐associated metabolites in HFD and LFD cells. H) Gene set enrichment analysis (GSEA) showing the top ten enriched pathways in HFD cells from RNA‐sequencing data in HFD and LFD cells (upper panel). Lipid metabolism‐associated pathways are depicted in red, and GSEA‐enrichment plots are shown (lower panel). Data are expressed as the mean ± SD of at least three replicates. * *p* < 0.05, ** *p* < 0.01, *** *p* < 0.001, as determined by an unpaired two‐tailed Student's *t*‐test. ns, nonsignificant.

Given that glycolytic intermediates were increased in HFD cells (Figure 1J,K), we examined whether HFD tumor cells relied on glycolysis. Surprisingly, restriction of the glucose concentration (2.5 × 10^−3^ m) had a greater impact on the growth of LFD cells than on HFD cells (Figure S2A, Supporting Information). Assessment of the basal level of energy metabolism revealed that HFD cells exhibited a higher ratio of oxygen consumption rate (OCR) to extracellular acidification rate (ECAR) (**Figure** **2**A and Figure S2B, Supporting Information), suggesting that HFD cells preferentially utilized oxidative phosphorylation (OXPHOS) but not glycolysis metabolism. Measurement of the OCR further revealed that the capacities for mitochondrial respiration, including maximal and spare respiration, were significantly increased in HFD cells (Figure 2B). To clarify the energy dependency of mitochondrial metabolism in HFD and LFD tumor cells, specific inhibitors of glycolysis, FAO, and glutaminolysis were administered. Results showed that blocking of glycolysis exhibited greater growth inhibition toward LFD tumor cells compared to HFD tumor cells (Figure 2C). These data were similar to those by glucose restriction (Figure  S2A). In particular, inhibition of FAO significantly suppressed growth of HFD tumor cells, whereas it had no effect on LFD tumor cells. Inhibition of glutaminolysis had less of an effect on both HFD and LFD tumor cells (Figure 2C). These data suggest that lipid metabolism might provide an additional source of energy supply for HFD tumor cells. As we expected, the addition of palmitate increased FAO activity in HFD cells, compared to LFD cells (Figure 2D). Accordingly, inhibition of FAO by etomoxir substantially suppressed invasion and tumorsphere formation in HFD, but not in LFD tumor cells (Figure 2E), indicating that HFD cells may take advantage of FAO. Indeed, lipidomics analysis revealed higher FAO intermediate metabolites in HFD tumor cells than in LFD cells (Figure 2F). Moreover, intermediates of the citric cycle were substantially elevated in HFD cells (Figure 2G) and we also found the upregulations of malate‐aspartate shuttle and reduced nicotinamide adenine dinucleotide (NADH) to nicotinamide adenine dinucleotide (NAD+) ratio in HFD cells, implicating the increased capacity of mitochondrial oxidative phosphorylation (Figure S2C, Supporting Information). RNA‐sequencing further revealed that upregulated gene sets in HFD tumor cells were related to cholesterol homeostasis, oxidative phosphorylation, and fatty acid metabolism (Figure 2H). These data confirmed a metabolic shift to FAO in obesity‐associated breast tumor cells.

### 2.3 YAP Signaling Is Regulated by FAO in Obesity‐Associated Breast Tumor Cells

**Figure 3:**
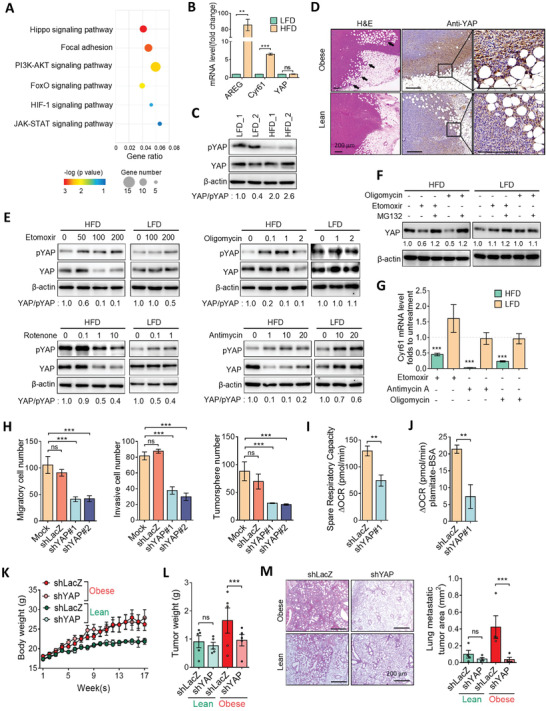
Upregulation of YAP in obesity‐associated breast cancer. A) Kyoto Encyclopedia of Genes and Genomes (KEGG) analysis of upregulated pathways in HFD from RNA‐sequencing data in HFD and LFD cells. The top six signaling pathways enriched in HFD cells are shown. B) qPCR analysis of YAP and downstream gene expressions in HFD and LFD cells. C) Western blot analysis of total and phosphorylated YAP (pYAP) levels in HFD and LFD cells. Fold changes expression of YAP to pYAP were quantified by using ImageJ software. D) H&E images of mice tumor sections (left panel). Immunohistochemistry and immunofluorescent images of YAP protein expression in tumor tissues from obese and lean mice. Enlarged pictures are shown in the right panel. Scale bar = 200 µm. E) Total and phosphorylated YAP protein levels of HFD and LFD cells treated with etomoxir, rotenone, antimycin, or oligomycin for 24 h. F) Western blot analysis of YAP protein level in response to MG132 (20 × 10^−6^ m), oligomycin (10 × 10^−6^ m), and etomoxir (100 × 10^−6^ m) for 12 h. G) qPCR analysis of Cyr61 gene expressions in HFD and LFD cells treated with etomoxir, rotenone, antimycin, or oligomycin for 24 h. H) The migration, invasion, and tumorsphere‐formation abilities of HFD/shcontrol and HFD/shYAP cells. I) Measurements of the spare respiration capacity from the oxygen consumption rate (OCR) and J) FAO dependency in HFD/shcontrol and HFD/shYAP cells with a Seahorse Bioanalyzer. The Δ oxygen consumption rate (OCR) was calculated as the OCR at the time of the BSA‐conjugated palmitate injection. K–M) Knockdown of YAP inhibits obesity‐promoted breast tumorigenesis. Obese and lean mice were inoculated with Py8119/shLacZ and shYAP cells for 8 weeks, and K) mice body weights and L) tumor weights were measured (*n *= 5/group). M) H&E images of mice lung sections (*n *= 4/group), quantitative data of metastatic tumor areas are shown in the right panel. Scale bar = 200 µm. Data are expressed as the mean ± SD of at least three replicates. * *p* < 0.05, ** *p* < 0.01, *** *p* < 0.001, as determined by an unpaired two‐tailed Student's *t*‐test. ns, nonsignificant.

To identify oncogenic signaling events associated with metabolic reprogramming in HFD tumor cells, we revisited upregulated genes from RNA sequencing, and gene ontology analysis revealed that enriched pathways included phosphoinositide 3‐kinases (PI3K)‐protein kinase B (AKT), FoxO, hypoxia‐inducible factor‐1, and Janus kinase‐signal transduction and activator of transcription in HFD tumor cells (**Figure** **3**A). Among these, the Hippo/YAP signaling was the most significantly enriched pathway (Figure 3A and Figure S3A, Supporting Information). To validate the involvement of Hippo signaling in HFD tumor cells, we analyzed expressions of YAP and YAP downstream targets. Results of the real‐time quantitative polymerase chain reaction (RT‐qPCR) analysis showed that YAP downstream targets, including *Amphiregulin* (*AREG*) and *Cyr61*, were substantially upregulated in HFD tumor cells, but the mRNA level of YAP showed no significant difference between HFD and LFD cells (Figure 3B). Western blot analysis further revealed that total YAP protein levels did not significantly change between LFD and HFD cells, but phosphorylated YAP was decrease in HFD cells, compared to LFD cells (Figure 3C). Moreover, histological examination showed the upregulation of YAP in HFD tumor tissues, specifically in the invasive front which is associated with adipocytes (Figure 3D and Figure S3B, Supporting Information). Similar to our findings, gene set enrichment analysis (GSEA) data revealed that YAP and lipid metabolism signatures were positively correlated with obese‐isolated E0771 cells than that in the chow diet group (Figure S3C, Supporting Information), suggesting that YAP and lipid metabolism may play an essential role in obesity‐associated breast tumorigenesis. Because YAP signaling can be modulated by energy stress,^[^ ^13^ ^]^ we investigated the effect of FAO on YAP regulation. HFD and LFD cells were treated with the FAO inhibitor, etomoxir, and results showed that etomoxir robustly increased YAP phosphorylation accompanied by decreased YAP protein levels in HFD cells (Figure 3E). In contrast, LFD tumor cells were less affected by inhibition of FAO (Figure 3E). Likewise, inhibition of electron transport chain (ETC) complexes by rotenone (complex I), antimycin (complex III), and oligomycin (complex V) reduced the YAP‐to‐phosphorylated‐YAP ratio in HFD tumor cells by greater magnitudes compared to those in LFD cells (Figure 3E). Accordingly, inhibition of FAO decreased YAP protein level, but that was restored by the proteosomal inhibitor, MG132. These phenomenon was observed in HFD but not LFD cells (Figure 3F), suggesting that increased FAO promotes YAP function via reducing YAP phosphorylation and increasing protein stability. Moreover, inhibition of FAO or ETC significantly downregulated Cyr61 expression in HFD but not LFD tumor cells (Figure 3G). To evaluate the effect of YAP on HFD tumor cells, we knocked‐down HFD cells with short hairpin YAP, and silencing of YAP markedly suppressed invasion and tumorsphere formation (Figure 3H and Figure S4A, Supporting Information). Moreover, the mitochondrial respiratory capacity and FAO activity were significantly reduced upon YAP inhibition (Figure 3I,J). To confirm the importance of YAP in obesity‐mediated breast tumor progression, parental Py8119 cells with YAP silencing were inoculated into obese and lean mice. Results showed that suppression of YAP created no differences in mouse body weight changes (Figure 3K and Figure S4B,C, Supporting Information). Importantly, YAP silencing significantly alleviated the tumor burden and lung metastasis in obese mice, but it had a modest effect in the lean group (Figure 3L,M). These findings indicated that obesity induces a metabolic shift to FAO, which in turn, activates YAP and facilitates breast tumor development.

### 2.4 YAP Governs the Antioxidant Capacity and Mitochondrial Redox Homeostasis

**Figure 4:**
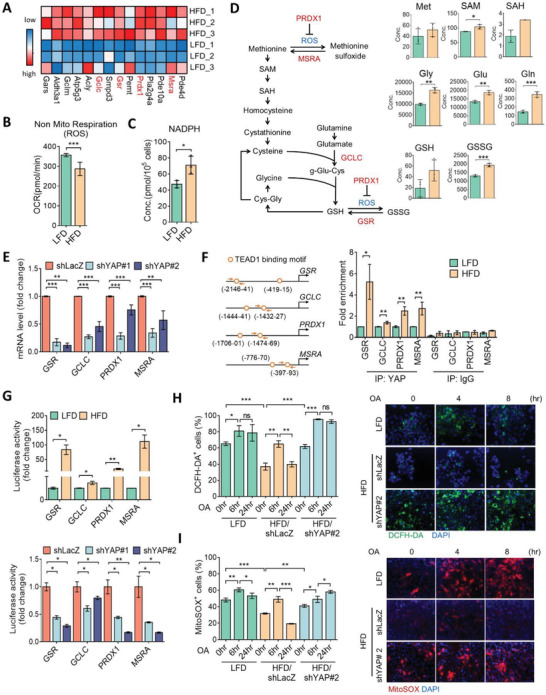
YAP governs mitochondrial oxidative stress in obesity‐associated tumor cells. A) Heatmap of differentially expressed metabolic genes associated with YAP in HFD and LFD cells. B) The nonmitochondrial respiratory ROS production in LFD or HFD cells with a Seahorse Analyzer. C) Absolute quantitative analysis of NADPH level in HFD and LFD cells. D) Changes in the concentrations of metabolites in glutathione metabolism in HFD and LFD cells. E) qPCR analysis of MSRA, GCLC, GSR, and PRDX1 expressions in HFD/shLacZ and HFD/shYAP cells. F) Illustration of putative TEAD1‐binding sites in the MSRA, GCLC, GSR, and PRDX1 promoter regions (left panel). Arrows indicate primers for the chromatin immunoprecipitation analysis. Enrichment of YAP in the MSRA, GCLC, GSR, and PRDX1 promoters by chromatin immunoprecipitation assay (right panel). G) Luciferase reporter assay of the transcriptional activities of MSRA, GCLC, GSR, and PRDX1 in LFD and HFD tumor cells (upper panel) and in HFD/shLacZ and HFD/shYAP cells (lower panel). H) Flow cytometric analysis of 2'‐7'dichlorofluorescin diacetate (DCFH‐DA+) cell populations in LFD, HFD/shLacZ, and HFD/shYAP cells exposed to oleic acid (OA: 100 × 10^−6^ m) for 0, 6, and 24 h, and representative images by fluorescent microscope observations are shown in the right panel. I) Flow cytometric analysis (left panel) and fluorescent images (right panel) of MitoSOX+ cells in LFD, HFD/shLacZ, and HFD/shYAP cells exposed to OA as described above. Data are expressed as the mean ± SD of at least three replicates. * *p* < 0.05, ** *p* < 0.01, *** *p* < 0.001, as determined by an unpaired two‐tailed Student's *t*‐test. ns, nonsignificant.

To further identify the molecular events triggered by YAP in HFD tumors, we focused on metabolism‐related genes that were upregulated in HFD tumors and might be transcriptionally controlled by YAP through an in silico analysis. Interestingly, glutathione redox‐ and antioxidant enzyme‐associated genes, including *glutamate‐cysteine ligase catalytic subunit* (*GCLC*), *glutathione‐disulfide reductase* (*GSR*), *peroxiredoxin 1* (*PRDX1*), and *methionine sulfoxide reductase A* (*MSRA*), were significantly enriched in HFD tumors (**Figure** **4**A). GCLC and GSR participate in glutathione biosynthesis, and PRDX1 and MSRA play roles reducing protein peroxidation. These findings drew our attention to the aforementioned data of decreased nonmitochondrial ROS by OCR (Figures 2B and 4B), and increased cellular reducing cofactor, nicotinamide adenine dinucleotide phosphate (NADPH), in HFD tumor cells (Figure 4C). Moreover, the concentrations of glutathione dependent metabolism and detoxification intermediates were upregulated in HFD cells (Figure 4D). These suggest a possible link between YAP and redox homeostasis in obesity‐associated tumors. To address this, HFD tumor cells were knocked‐down with YAP, and an RT‐qPCR analysis identified downregulation of GCLC, GSR, PRDX1, and MSRA mRNA levels upon YAP inhibition (Figure 4E). The ChIP assay further revealed tentative YAP/TEAD1‐binding sites and validated the enhanced bindings of YAP in promoter regions of antioxidant genes in HFD tumor cells than in LFD cells (Figure 4F). Moreover, the promoter activities of GCLC, GSR, PRDX1, and MSRA were significantly elevated in HFD tumor cells compared to LFD cells (Figure 4G). In contrast, knockdown of YAP in HFD cells significantly suppressed transcriptional activities of GCLC, GSR, PRDX1, and MSRA (Figure 4G).

To validate the contribution of the YAP‐mediated antioxidant capacity, the intracellular ROS content was measured with DCHF‐DA fluorescent dye. As shown in Figure 4H, the basal intracellular ROS level was lower in HFD cells compared to LFD cells. Conversely, silencing of YAP in HFD cells recapitulated the ROS increase (Figure 4H). Interestingly, treatment with oleic acid (OA) induced ROS productions with 6 h of incubation in both HFD and LFD cells, but the increased ROS level was diminished after 24 h of incubation only in HFD cells. This phenomenon was not observed in either LFD or HFD/shYAP cells (Figure 4H). Similar results were detected by staining with the mitochondrial ROS dye, MitoSOX (Figure 4I), validating the involvement of mitochondrial ROS and the function of YAP in mitigating oxidative stress in mitochondria. These data support the notion that YAP is a key regulator of mitochondrial redox homeostasis.

### 2.5 YAP Is an ROS Sensor and Overcomes Obesity‐Associated Oxidative Stress

**Figure 5:**
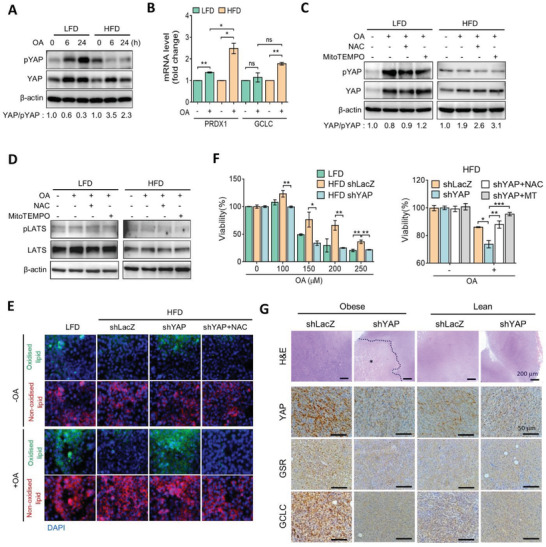
YAP extenuates fatty acid‐mediated oxidative stress. A) Total and phosphorylated YAP protein levels of HFD and LFD cells treated with OA for 6 or 24 h, as assessed by Western blotting. B) qPCR analysis of *GCLC* and *PRDX1* gene expressions in HFD and LFD cells treated with OA for 24 h. C) OA (100 × 10^−6^ m) was added to HFD or LFD cells pretreated with *N*‐acetyl‐cysteine (NAC) (2 × 10^−3^ m) or MitoTEMPO (5 × 10^−6^ m) for 24 h, and protein levels of total and phosphorylated YAP were assessed by Western blotting. D) OA (100 × 10^−6^ m) was added to HFD or LFD cells pretreated with NAC (2 × 10^−3^ m) or MitoTEMPO (5 × 10^−6^ m) for 6 h, and protein levels of total and phosphorylated LATS1 were assessed by Western blotting. E) LFD, HFD, or HFD shYAP cells were treated with OA for 24 h and stained with BODIPY 581/591 C11. F) LFD, HFD shLacZ, and shYAP cells were treated with different concentrations of OA for 24 h. OA (125 × 10^−6^ m) was added in HFD shLacZ and shYAP cells pretreated with NAC (2 × 10^−3^ m) or MitoTEMPO (5 × 10^−6^ m) for 24 h. Cell viability was measured by an 3‐(4,5‐Dimethylthiazol‐2‐yl)‐2,5‐diphenyltetrazolium bromide (MTT) assay. G) H&E and IHC images of YAP, GSR, and GCLC protein expressions in tumor tissues from obese and lean mice inoculated with Py8119/shLacZ and shYAP cells. Asterisk denotes necrotic area. Scale bar = 200 µm (H&E panels) and 50 µm (IHC panels). Data are presented as the mean ± SD. * *p* < 0.05, ** *p* < 0.01, *** *p* < 0.001, as determined by an unpaired two‐tailed Student's *t*‐test. ns, nonsignificant.

We next explored the role of YAP‐mediated redox homeostasis in metabolic adaptation and survival advantages. HFD and LFD cells were treated with oleic acid. As shown in **Figure** **5**A, treatment with oleic acid increased the YAP‐to‐phosphorylated‐YAP ratio in HFD cells. The YAP protein level was elevated in both HFD and LFD cells in response to oleic acid; however, phosphorylated YAP was elevated only in LFD not in HFD cells (Figure 5A). Results of the RT‐qPCR also showed that oleic acid induced a greater magnitude of antioxidant gene expressions in HFD cells, compared to that in LFD cells (Figure 5B). We hypothesized that the elevated antioxidant capacity in HFD cells may be associated with YAP signaling activation. In LFD cells, we found that oleic acid‐induced phosphorylation of YAP was impeded by the antioxidant, *N*‐acetyl‐cysteine (NAC), and the mitochondrion‐specific superoxide scavenger, MitoTEMPO (Figure 5C). However, phosphorylation of YAP showed less alteration regardless of oleic acid or antioxidant treatments in HFD cells (Figure 5C). Similar patterns were found in the total YAP protein (Figure 5C), suggesting that YAP is sensitive to mitochondrial ROS. We also investigated the upstream regulator of YAP, LATS1. Results showed that oleic acid failed to induce phosphorylation of LATS1 in either LFD or HFD cells (Figure 5D), suggesting that mitochondrial ROS trigger phosphorylation of YAP in a Hippo‐independent manner.

To validate whether the YAP‐regulated antioxidant capacity participates in alleviating intracellular oxidative stress, cells were stained with the lipid peroxidation probe, BODIPY‐C11, in response to oleic acid. As shown in Figure 5E, oleic acid robustly induced lipid peroxidation in LFD cells, compared to HFD cells. However, suppression of YAP resulted in increasing lipid peroxidation in HFD cells, which was diminished by NAC (Figure 5E). Moreover, HFD cells showed more tolerance to fatty acid‐mediated growth inhibition compared to LFD cells, and suppression of YAP in HFD cells increased sensitivity to fatty acid treatment (Figure 5F). However, treatment with NAC recapitulated the increased ROS in YAP‐knockdown cells (Figure 5F). These data suggest that the YAP‐mediated antioxidative capacity renders an ability to ameliorate lipid peroxidation. Furthermore, histological examinations showed that levels of YAP, GSR, and GCLC proteins were concomitantly elevated in Py8119 tumor tissues from obese mice, compared to those in lean mice (Figure 5G), but increased expressions of YAP, GSR, and GCLC were reduced by YAP‐knockdown (Figure 5G). We also noted that suppression of YAP resulted in increased necrotic areas in obesity‐associated tumor tissues (Figure 5G). These data indicated that YAP governs mitochondrial redox homeostasis to overcome obesity‐related oxidative stress.

### 2.6 Adipocytes Fuel Breast Tumor Cells via the FAO‐AMPK‐YAP Signaling Axis

**Figure 6:**
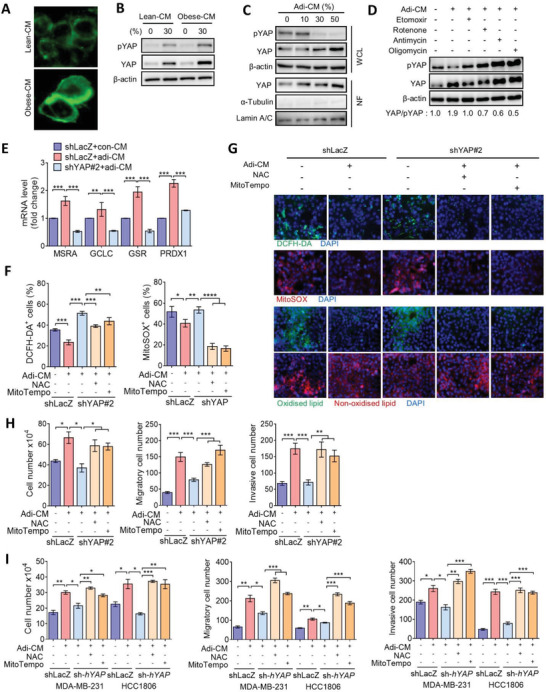
Adipocytes promote aggressiveness of TNBC in a YAP‐driven antioxidant manner. A) BODIPY staining of lipid accumulation in Py8119 cells incubated with lean‐CM or obese‐CM for 24 h. B) Western blot analysis of total and phosphorylated YAP levels in Py8119 cells treated with lean‐CM or obese‐CM. C) Western blot analysis of total and phosphorylated YAP levels in the whole‐cell lysate (WCL) and total YAP levels in the nuclear fraction (NF) of Py8119 cells treated with different concentrations of adi‐CM. D) Py8119 cells were pretreated with etomoxir, rotenone, antimycin, or oligomycin and then incubated with adi‐CM for 24 h. Expressions of YAP and phosphorylated YAP were assessed by Western blotting. E) qPCR analysis of *MSRA*, *GCLC*, *GSR*, and *PRDX1* gene expressions in Py8119/shLacZ and shYAP cells exposed to adi‐CM. (F‐H) Py8119/shLacZ and shYAP cells were pretreated with NAC (2 × 10^−3^ m) or MitoTEMPO (5 × 10^−6^ m) followed by incubation with adi‐CM for another 24 h, F) DCFH‐DA+ and MitoSOX+ cells were analyzed by flow cytometry, G) fluorescent microscopic observations of DCFH‐DA, MitoSOX, and BODIPY 581/591 C11 staining, and H) cell proliferation and transwell migration and invasion assays were performed. I) MDA‐MB‐231/shLacZ, HCC1806/shLacZ, MDA‐MB‐231/shYAP, and HCC1806/shYAP cells were pretreated with NAC or MitoTEMPO followed by incubation with adi‐CM for 24 h. Cell proliferation and transwell migration and invasion assays were performed. Data are presented as the mean ± SD. * *p* < 0.05, ** *p* < 0.01, *** *p* < 0.001, as determined by an unpaired two‐tailed Student's *t*‐test.

Given that YAP expression was elevated in adipocytes surrounding tumor cells (Figure 3D), this suggests that a possible interaction between adipocytes and tumor cells may regulate YAP signaling. We isolated abdominal adipocytes from obese and lean mice, and adipocyte‐derived condition medium (CM) was used to treat parental Py8119 cells. Lipid staining showed that lipid droplets accumulated by obese‐adipocyte CM‐treated compared to lean‐adipocyte CM‐treated cells (**Figure** **6**A). Moreover, treatment with obese‐adipocyte CM substantially upregulated the YAP protein level (Figure 6B), suggesting that adipocytes‐derived fatty acids may contribute to induce YAP activation for breast cancer cells. To address this, Py8119 cells were treated with CM from mature 3T3L1 adipocytes (adi‐CM) (Figure S5A,B, Supporting Information). Similar to the above findings, treatment of Py8119 cells with adi‐CM increased lipid droplet accumulation (Figure S5C, Supporting Information) accompanied by decreased phosphorylation of YAP and an increased total YAP protein and nuclear translocation of YAP (Figure 6C). However, expression patterns of the YAP‐to‐phosphorylated‐YAP ratio decreased with pretreatment with inhibitors of FAO and ETC (Figure 6D). Accordingly, the growth and invasiveness of Py8119 cells were increased by treatment with adi‐CM (Figure S5D, Supporting Information) but not by control 3T3‐L1 CM‐treated cells (Figure S5C–E, Supporting Information), and YAP‐knockdown substantially diminished growth, invasiveness, and stemness of Py8119 cells in response to adi‐CM (Figure S5F–H, Supporting Information). Because YAP can be phosphorylated via AMP‐activated protein kinase (AMPK) in response to the energy status. Our data showed that adi‐CM reduced phosphorylation of AMPK which was accompanied by decreased YAP phosphorylation compared to con‐CM‐treated cells (Figure S6A, Supporting Information). However, the reductions in AMPK and YAP phosphorylation by adi‐CM were restored in the presence of etomoxir and metformin (Figure S6B, Supporting Information). Moreover, pretreatment of the inhibitor of the fatty acid transporter also reversed YAP phosphorylation (Figure S6C, Supporting Information). These data demonstrate that increased fatty acid uptake and oxidation are responsible for YAP activation, which is mediated by AMPK. Thus, adipocyte‐mediated YAP activation is mediated through energy metabolism‐dependent AMPK signaling.

We further investigated the contribution of the antioxidative capacity of breast tumor cells in response to adi‐CM. As shown in Figure 6E, the RT‐qPCR assay revealed that expressions of antioxidant enzymes were upregulated by adi‐CM, whereas that were suppressed upon silencing of YAP (Figure 6E). Additionally, treatment with adi‐CM reduced intracellular and mitochondrial ROS levels, and those were restored in YAP‐knockdown Py8119 cells (Figure 6F,G). However, the increased ROS levels in YAP‐knockdown cells were impeded by NAC and mitoTempo (Figure 6F,G). Consistent results were observed using BODIPY‐C11 staining (Figure 6G). Moreover, treatments with NAC and mitoTempo recapitulated the inhibition of growth and invasiveness in YAP‐silenced cells (Figure 6H). These data indicate that YAP‐regulated redox homeostasis plays a crucial role in adipocyte‐promoted breast tumorigenesis.

To further address the importance of YAP in the redox balance in human breast cancer cells, we treated TNBC MDA‐MB‐231 and HCC1806 cells with adi‐CM. Consistent with the aforementioned findings, treatment of MDA‐MB‐231 cells with adi‐CM increased the YAP‐to‐phosphorylated‐YAP pattern (Figure S7A, Supporting Information) and promoted the proliferation, migration, and invasion of human TNBC cells (Figure 6I and Figure S7B,C, Supporting Information). However, adi‐CM‐promoted growth and invasiveness were suppressed by YAP silencing (Figure 6I and Figure S7B,C, Supporting Information), but they were rescued by the addition of NAC and mitoTempo (Figure 6I). Consistent results were also obtained in estrogen receptor (ER+) MCF7 cells (Figure S8A–D, Supporting Information). These data together indicated that FAO triggers YAP activation in both ER+ and ER− breast cancer cells and highlighted that YAP‐regulated antioxidant plays a critical role in obesity‐driven breast tumorigenesis.

**Figure 7:**
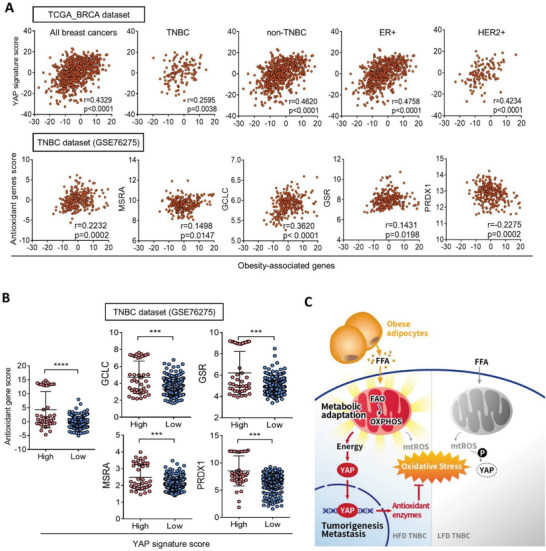
Association of YAP and antioxidant genes in breast cancer patients. A) Pearson correlation of YAP signature scores and obesity‐associated genes in triple‐negative, nontriple‐negative, HER2+, ER+, and all breast cancers in TCGA database (upper panel). Pearson correlations of antioxidant gene scores and obesity‐associated genes in TNBC patients from GSE76275 dataset (lower panel). B) The antioxidant gene score in TNBC patients from the GSE76275 dataset with low and high YAP signature scores. Data are presented as the mean ± SD. * *p* < 0.05, ** *p* < 0.01, *** *p* < 0.001, as determined by an unpaired two‐tailed Student's *t*‐test. C) Schematic diagram summarizing the proposed model for the mechanism of metabolic adaptation in obesity‐associated breast cancer. Adipocytes induce a metabolic shift to FAO in obesity‐associated breast tumor cells, which subsequently activates YAP and enhances the antioxidant capacity to protect against mitochondrial oxidative stress. Contrarily, fatty acids increased ROS levels and decreased YAP stability, resulting in elevating lipid peroxidation in LFD cells. The metabolic adaptation driven by YAP renders capabilities to ameliorate metabolic stress and tumor propagation.

Finally, we analyzed the association between YAP and antioxidant genes in breast cancer patients. Data revealed that obese‐associated gene expressions showed positive correlations with the YAP signature across different subtypes of breast cancer (**Figure** **7**A), and positive correlations between obesity‐associated genes and the antioxidant score were found in TNBC patients (Figure 7A). Moreover, TNBC patients with higher YAP signatures were associated with higher antioxidant gene patterns (Figure 7B). Accordingly, the in vitro and clinical analyses showed that breast cancer with elevated antioxidant gene expressions were associated with poorer responses to conventional chemotherapies (Figure S9, Supporting Information). Together, these findings indicated that the YAP‐mediated antioxidative capacity plays a crucial role in breast cancer development and it confers clinical impacts of the YAP‐antioxidant axis in obesity‐associated breast cancer.

## 3 Discussion

The metabolic reprogramming to glycolysis is a well‐established hallmark of cancer; however, tumor cells may rely on distinct metabolic pathways, depending on different metabolic microenvironments. This flexibility renders tumor cells able to utilize energy resources to adapt to fluctuating conditions. YAP is an energy sensor and was reported to participate in regulating a variety of metabolic processes,^[^ ^14^ ^]^ thereby drawing much attention to cancer metabolism. Our previous study and studies by others showed that overexpression of YAP regulated the glucose transporter and promoted glycolysis in metastatic colorectal and brain tumor cells.^[^ ^15^ ^]^ A recent study reported that activation of YAP enhanced FAO activity in lymph node metastatic tumor cells;^[^ ^16^ ^]^ however, the contribution of YAP to this adaptation toward FAO is still unclear. In the present study, using a comparative analysis of obese‐ and lean‐associated tumor tissues in conjunction with metabolomics and transcriptomics, we found a global metabolic switch in obesity‐associated tumor cells. We identified that YAP is a key driver for dispelling FAO‐elicited oxidative stress in obesity‐associated tumor cells. YAP regulates antioxidant gene expressions and controls mitochondrial redox homeostasis. Although the in silico analysis did not observe YAP to be involved in either FAO‐ or OXPHOS‐related gene regulation in our model, these data were similar to a recent study which reported that TEAD4 modulated OXPHOS complex expressions independent of YAP.^[^ ^17^ ^]^ We found that the transcriptional level of YAP was not significantly changed between HFD and LFD cells. However, the phosphorylated YAP level was decreased in HFD cells. Blocking of FAO and ETC suppressed YAP to phosphorylated‐YAP pattern preferentially in HFD. Moreover, inhibition of FAO decreased YAP protein level but that was restored by the proteosomal inhibitor. These data indicate that HFD cells are addicted to FAO, which promotes YAP protein stability. These data were similar with previous findings that YAP phosphorylation can be regulated by energy stress.^[^ ^13a^ ^]^ Importantly, we showed for the first time that YAP regulated antioxidant genes expression, while phosphorylation of YAP could be attenuated by antioxidants, indicating that YAP‐mediated redox balance is beneficial to increase its protein stability. Thus, this mechanism not only facilitates adjustment to the FAO pathway but also renders tumor cells capable of resisting oxidative stress. Therefore, YAP governs redox homeostasis by adapting to obesity‐mediated metabolic stress thereby promoting survival advantage (Figure 7C).

Accumulating evidence suggests that highly malignant and stem‐like subpopulations of tumor cells exhibit a distinct metabolic shift from glycolysis to OXPHOS.^[^ ^18^ ^]^ Increased OXPHOS is concomitantly associated with ROS levels. Intracellular ROS not only damage cellular functions but also elicit signaling transduction in another fashion, indicating that these subpopulations of cells may alter their antioxidant capacity to maintain a redox balance.^[^ ^19^ ^]^ A recent study reported that gastric cancer stem cells exhibited decreased mitochondrial ROS levels and increased drug resistance through peroxiredoxin 3 (PRDX3) expression.^[^ ^20^ ^]^ In agreement with this, we identified that obesity‐associated tumor cells were associated with reduced mitochondrial ROS contents. Moreover, we identified that YAP regulated expressions of antioxidant enzymes involved in glutathione biosynthesis (GSR and GCLC) and protein peroxidation (PRDX1 and MSRA). Suppression of YAP in obesity‐associated tumor cells increased mitochondrial ROS concentrations and failed to scavenge fatty acid‐mediated lipid peroxidation. Interestingly, we also found that mitochondrial ROS triggered YAP phosphorylation, possibly in a Hippo‐independent manner. It was recently reported that hydrogen peroxide inhibited tumor mortality by phosphorylating MST1, an upstream regulator of LATS1 and YAP. However, inhibition of YAP had less effect on hydrogen peroxide‐mediated inhibition of tumor mortality, suggesting a YAP‐independent, noncanonical Hippo pathway linking oxidative stress and inhibition of tumor metastasis.^[^ ^21^ ^]^ Our study showed that FAO activated YAP, and in turn, mitigated mitochondrial oxidative stress. We also found that YAP phosphorylation was regulated by mitochondrial ROS. Accordingly, an elevated obesity signature in breast cancer patients was correlated with YAP and antioxidant genes, and higher expressions of antioxidant genes conferred a poor response to chemotherapies. It is interesting to note that glutathione metabolism plays an essential role in ferroptosis, which participates in acquired resistance to anticancer therapeutics. These findings highlight the crucial role of YAP in dictating obesity‐driven metabolic switch and cellular redox homeostasis, and provide clinical aspects in a therapeutic approach. Interestingly, recent findings reported that YAP repressed ER*α* in ER+ breast cancer cells.^[^ ^22^ ^]^ Our data showed that treatment with adi‐CM for 24 h downregulated ER*α* expression in MCF7 cells (Figure S8E, Supporting Information). Additionally, we also found that expression of YAP (*YAP1*) was negatively correlated with ER*α* (*ESR1*) in ER+ breast cancer patients, but the *ESR1* level was still positively correlated with YAP‐ and obesity‐associated signatures (Figure S8F, Supporting Information). However, the correlation between *ESR1* and obesity was less significant (Pearson *r* = 0.225), compared to the association between YAP and obesity (Pearson *r* = 0.476) (Figure S8G, Supporting Information). These data highlighted that YAP signaling indeed play a critical role in obesity‐associated breast cancer. Although activation of YAP was identified to repress ER*α* transcription and inhibit cell growth in ER+ breast tumor cells, a recent study reported that YAP was elevated in tamoxifen‐resistant breast cancer, and overexpression of YAP led to downregulation of ER*α* expression and conferred resistance to antihormone therapy in ER+ breast cancer.^[^ ^23^ ^]^ Therefore, the role of YAP in ER+ breast cancer, and the contribution of YAP in obese‐related ER+ breast tumor progression is warranted to investigate.

Several previous studies reported that induction of metabolic reprogramming by adipocytes facilitates tumor progression,^[^ ^9^, ^24^ ^]^ the molecular function of YAP in the obesity‐mediated metabolic shift is still unknown. Herein, we found that YAP expression was elevated in the adipocyte‐surrounded tumor front, and we further identified that adipocyte CM‐mediated ROS reduction and tumor promotion in TNBC cells were dependent on YAP. Inhibition of FAO and OXPHOS impeded YAP activation when exposed to adi‐CM, indicating that metabolic cues are important for adipocyte‐mediated YAP activation, although we cannot preclude that obesity‐associated adipokines may participate in this regulatory mechanism. Our cytokine array data showed that several inflammatory factors were upregulated in adi‐CM, compared to the control‐CM (Figure S10, Supporting Information). These cytokines may regulate YAP signaling in a different manner. Therefore, the pro‐tumor action by adi‐CM might be close to the context of HFD cells in the obese tumor microenvironment. Interestingly, a recent study showed that adipocytes promoted prostate cancer cell invasion through stimulation of expression of the pro‐oxidant enzyme, NADPH oxidase, and ROS production; however, inhibition of FAO had less of an inhibitory effect on prostate cancer cell invasion.^[^ ^25^ ^]^ In contrast, inhibition of FAO substantially impeded breast cancer cell invasion in response to adipocytes,^[^ ^8a^ ^]^ indicating that a distinct metabolic pathway via the FAO pathway is preferentially utilized in obesity‐associated breast tumor cells. Additionally, Gao and colleagues recently reported that the homolog of YAP, transcriptional co‐activator with PDZ‐binding motif (TAZ)‐regulated resistin by adipocytes played a role in mediating breast tumorigenesis.^[^ ^26^ ^]^ Furthermore, activation of YAP/TAZ signaling was obligated to orchestrate tumor surrounding stromal cells.^[^ ^27^ ^]^ These data suggest a possible role of YAP in governing obese‐associated TME. Thus, it is worth investigating the role of YAP/TAZ‐mediated metabolic remodeling in the crosstalk between breast tumors and the TME.

Taken together, our findings not only provide a fundamental understanding of YAP in metabolic adaptation but also uncover a metabolic vulnerability in obesity‐elicited breast cancer development. This finding sheds light on the role of mitochondrial redox homeostasis in YAP‐driven tumorigenesis in obesity‐associated breast cancer.

## 4 Experimental Section

### Animal Study and Primary Cells Isolation

All animal studies were performed based on guidelines and approval of the Animal Care and Use Committee of Taipei Medical University (LAC2018‐0255). Female 5‐week‐old C57BL/6 mice (National Laboratory Animal Center, Taipei, Taiwan) were fed diets (Research Diets, Inc.) containing 10 kcal% fat/no sucrose low‐fat diet (lean mice) or 60 kcal% fat high‐fat diet (obese mice) for 12 weeks. Mice body weights were monitored twice a week. To measure fasting glucose levels, mice were starved for 6 h, and blood glucose levels were measured with a blood glucometer (Roche Diagnostics). Then mouse TNBC Py8119 cells (American type culture collection, ATCC CRL‐3278) were orthotopically injected into the mammary fat pad, and diets were continued for 4 weeks. Tumor volumes were calculated according to the equation: (width) x (length)^2^/2. On day 28 after tumor inoculation, the mice were sacrificed, and their lungs and tumor tissues were harvested for histological examination. To obtain HFD and LFD tumor cells, the primary tumor tissues from four individual mice were subsequently isolated from obese and lean mice and defined as high‐fat diet (HFD#1 and HFD#2) and low‐fat diet (LFD#1 and LFD#2) tumor cells, respectively. Tumor tissues were cut into small pieces and disassociated with a gentleMACS tumor dissociation kit (Miltenyi Biotec). Suspensions were treated with a red blood cell (RBC) lysis buffer to remove RBCs. Approximately 3 × 10^6^ isolated cells were seeded in 10 cm plates and incubated at 37 °C for further experiments. Most of the data were performed by HFD#1 and LFD#1 cells unless specified otherwise. To obtain adipocytes from obese and lean animals, visceral fat from obese and lean mice were collected, minced, and obtained using an adipocyte isolation kit (Miltenyi Biotec) according to the manufacturer's instructions.

### Cell Culture

Human MDA‐MB‐231 and HCC1806 TNBC cell lines, the mouse Py8119 TNBC cell line, and mouse 3T3‐L1 preadipocyte cells were purchased from the Bioresource Collection Research Center (Hsinchu, Taiwan) or ATCC. MDA‐MB‐231 and Py8119 cells were maintained in Dulbecco's modified Eagle medium (DMEM), and HCC1806 cell were cultured in Roswell Park Memorial Institute 1640 medium supplemented with 7% fetal bovine serum (FBS), 1% antibiotic‐antimycotic, and 1% Glutagro. 3T3‐L1 cells were cultured in DMEM supplemented with 10% calf serum, 1% antibiotic‐antimycotic, and 1% Glutagro. All cell culture supplements were purchased from Corning Costar, and cells were cultured at 37 °C in a humidified atmosphere of 5% CO_2_. For adipocyte differentiation, 10^5^ 3T3‐L1 cells were seeded in gelatin‐coated six‐well plates and cultured in DMEM supplemented with 10% FBS, 0.5 × 10^−3^ m 3‐isobutyl‐1‐methylxanthine, 1 × 10^−6^ m dexamethasone, 1 × 10^−6^ m rosiglitazone, and 10 µg mL^−1^ insulin (Sigma) for 4 d and then placed in DMEM supplemented with 10% FBS, 10 µg mL^−1^ insulin, and an oil mix (40 × 10^−6^ m sodium palmate, 80 × 10^−6^ m sodium oleate, and 0.6 × 10^−3^ m linoleic acid; Sigma‐Aldrich) for another 2 d. Lipid droplets in mature adipocytes were stained with an oil red solution. To collect conditioned medium (CM) from mature adipocytes (adi‐CM), and lean and obese adipocytes, cells were washed with serum‐free medium and replaced with DMEM containing 1% FBS for 48 h. Preadipocyte CM was collected from 3T3‐L1 cells incubated in DMEM/1% FBS for 48 h.

### Short Hairpin RNA

Briefly, short hairpin RNAs (shRNAs) against mouse (TRCN0000238432; TRCN0000238436) and human (TRCN0000107265) YAP were obtained from the National RNAi Core Facility (Academia Sinica, Taipei, Taiwan). HEK293T cells were cotransfected with pLKO.shRNA together with the pCMV‐∆R8.91 and pMDG plasmids. At 48 h post‐transfection, virus‐containing supernatants were collected, centrifuged, and added to target cells for another 48 h. Medium of transduced cells was replaced with fresh medium, and cells were stably selected by puromycin (5 µg mL^−1^) for 7 d as described in the previous studies.^[^ ^15b^ ^]^

### Seahorse Assay

The extracellular acidification rate (ECAR) and OCR were measured with a Seahorse XFe24 Flux Analyzer (Seahorse Bioscience). Cells were seeded in Seahorse plates overnight to reach 90% confluence. For the mito stress assay, 1 h before the measurement, culture medium was replaced with XF basal medium (Seahorse Bioscience), and the plates were placed into a 37 °C non‐CO_2_ incubator. The OCR was measured over time following an injection of 1 × 10^−6^ m oligomycin, 0.5 × 10^−6^ m carbonyl cyanide‐p‐trifluoromethoxyphenylhydrazone (FCCP), and 0.5 × 10^−6^ m rotenone (Sigma). The maximal respiration and spare respiration capacities were respectively calculated as (the maximum rate measured after FCCP injection − the minimum rate measured after rotenone injection) and [maximal respiration—(the last rate measured before the first injection − the minimum rate measured after the rotenone injection)]. For the FAO activity assay, the culture medium was replaced with substrate‐limited growth medium for 4 h, the medium was replaced with XF basal medium (Seahorse Bioscience) and plates were placed in a 37 °C non‐CO_2_ incubator for 1 h. The OCR was measured over time following an injection of 1 × 10^−3^ m bovine serum albumin (BSA)‐conjugated palmitate. Levels of FAO activity were calculated as the ΔOCR = (the maximum rate measured after the palmitate‐BSA injection − the last rate measured before the first injection).

### Metabolomics, Cell Viability, Reverse Transcription and RT‐qPCR, Western Blotting, Luciferase Reporter Assay, Chromatin Immunoprecipitation (ChIP) Assay, Immunofluorescence, and Immunohistochemical (IHC) Analyses

Detailed methodologies of metabolomics, cell viability, RT‐qPCR, Western blotting, luciferase reporter assay, ChIP assay, and IHC analyses are shown in the “Materials and Methods” section in the Supporting Information. Specific primers for the RT‐qPCR are listed in Table S1 in the Supporting Information.

### Colony Formation, Tumorsphere Formation, and Transwell Assays

The methodologies of colony formation, tumorsphere formation, and transwell analyses were described in previous studies.^[^ ^15^, ^28^ ^]^

### Bioinformatics and Statistical Analyses

Gene expression patterns in the Cancer Genome Atlas breast cancer cohort (TCGA_BRCA) dataset were downloaded from the University of California, Santa Cruz Xena browser (https://xenabrowser.net/ accessed on 5 May 2021) and Gene Expression Omnibus GSE76275 database.^[^ ^29^ ^]^ The YAP signature score was calculated as the sum of the normalized value of expressions of YAP‐associated genes based on YAP conserved signature in the GSEA geneset (https://www.gsea‐msigdb.org). There are 19 obesity‐associated genes reported in the literature and the sum of the normalized value was calculated. A correlation coefficient was analyzed by the Pearson test. Data are presented as the mean ± standard error of three independent experiments (*n *= 3). Statistical significance was determined by an unpaired, two‐tailed Student's *t*‐test unless stated otherwise. Statistical significance was indicated as follows: * *p* < 0.05; ** *p* < 0.01; *** *p* < 0.001. Statistical analyses were carried out with GraphPad Prism 6.0 software.

## Conflict of Interest

The authors declare no conflict of interest.

## Author Contribution

The author contribution is as follows: study design and conduct (J.‐Z.D., C.‐W.L.); data collection (J.‐Z.D., Y.‐R.W., C.‐H.C., I‐L.T., Y.‐C.C.); data analysis and interpretation (J.‐Z.D., Y.‐R.W., C.‐H.C.); drafting paper (J.‐Z.D., C.‐W.L.); revising paper content and approving the final version of paper (C.‐W.L.).

## Supporting information

Supporting Information

## Data Availability

The data that support the findings of this study are openly available in YAP dictates mitochondrial redox homeostasis to facilitate obesity‐associated breast cancer progression at https://doi.org/[doi], reference number 41.
