# Supplementary material for: YAP Dictates Mitochondrial Redox Homeostasis to Facilitate Obesity‐Associated Breast Cancer Progression
Source: Adv Sci (Weinh). 2022 Feb 18;9(12):2103687. doi: 10.1002/advs.202103687 (PMC9035999; doi:10.1002/advs.202103687)
Supplement: Supplementary file 1 — Supporting Information [file ADVS-9-2103687-s001.pdf]

ADVANCED SCIENCE
Open Access

Supporting Information

for *Adv. Sci.*, DOI 10.1002/advs.202103687

YAP Dictates Mitochondrial Redox Homeostasis to Facilitate Obesity-Associated Breast Cancer Progression

*Jia-Zih Dai, Yen-Ju Wang, Cheng-Hsun Chen, I-Lin Tsai, Yi-Chun Chao and Cheng-Wei Lin**

Supporting Information for:

YAP dictates mitochondrial redox homeostasis to facilitate obesity-associated breast cancer progression

Jia-Zih Dai[1,2], Yen-Ju Wang[1,2], Cheng-Hsun Chen[1], I-Lin Tsai[1,2], Yi-Chun Chao[1,2], Cheng-Wei Lin[1,2,3,4#]

[1]*Department of Biochemistry and Molecular Cell Biology, School of Medicine, College of Medicine, Taipei Medical University, Taipei 110, Taiwan.*

[2]*Graduate Institute of Medical Sciences, College of Medicine, Taipei Medical University, Taipei 110, Taiwan.*

[3]*Cell Physiology and Molecular Image Research Center, Wan Fang Hospital, Taipei Medical University, Taipei 116, Taiwan.*

[4]*Drug Development and Value Creation Research Center, Kaohsiung Medical University, Kaohsiung 807, Taiwan.*

[#]**Corresponding author:** Cheng-Wei Lin; Department of Biochemistry and Molecular Biology, School of Medicine, College of Medicine, Taipei Medical University, 250 Wu-Xing Street, Taipei 11031, Taiwan. Email: cwlin@tmu.edu.tw; Phone: 886-2-27361661 ext. 3160; Fax: 886-2-27356689.

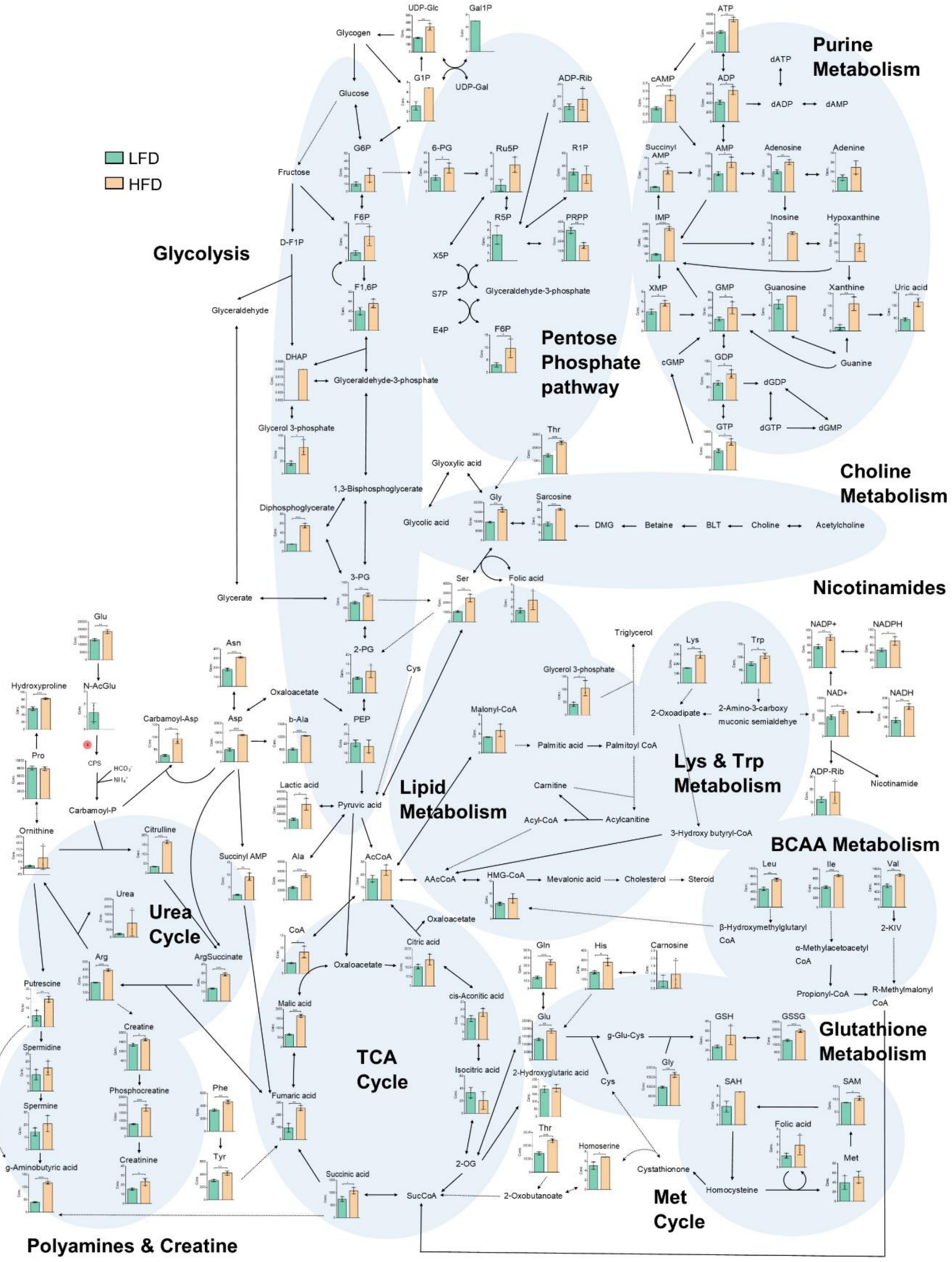

Supplementary Fig. 1 Metabolic reprogramming in HFD cells. Absolute quantitative analysis of metabolites of the central metabolic pathways in HFD or LFD cells. Data are presented as the mean ± SD. * *p*<0.05, ** *p*<0.01, *** *p*<0.001.

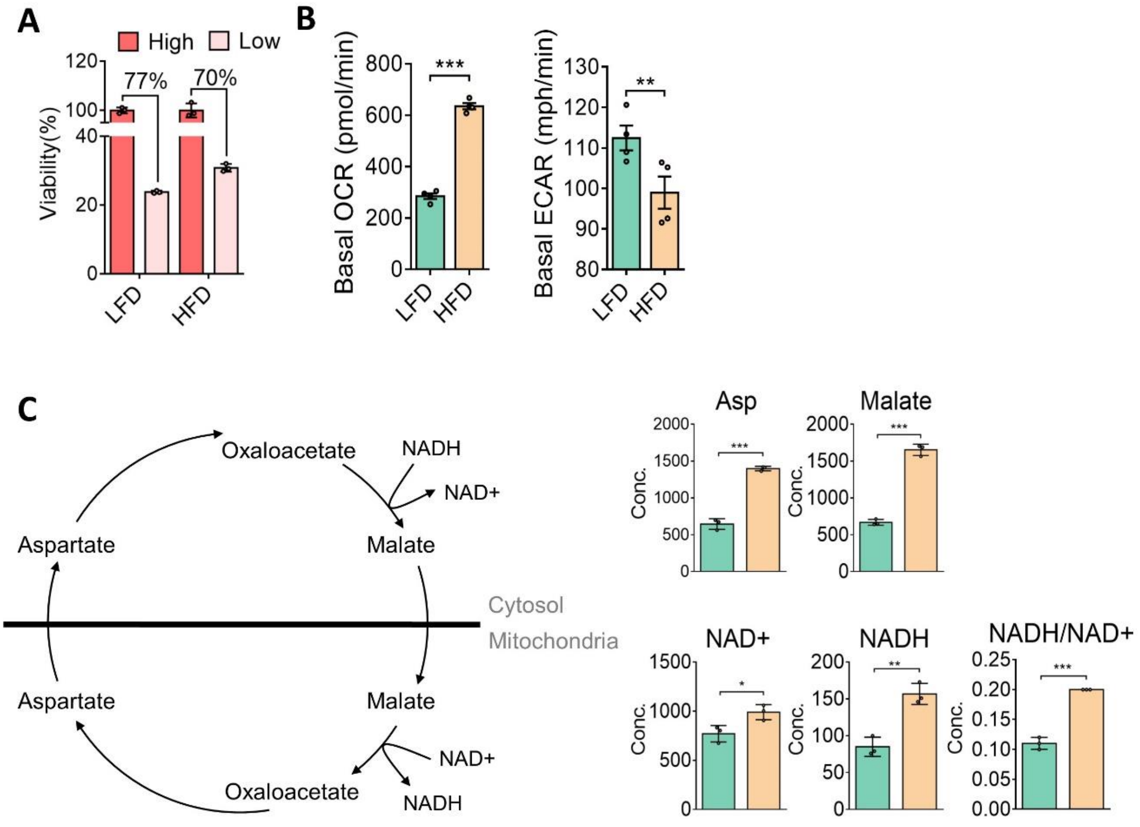

Supplementary Fig. 2 HFD cells rely on oxidative phosphorylation. (A) HFD and LFD cells were incubated with high (25 mM) or low (2.5 mM) concentrations of glucose-containing medium for 72 h, and cell viability was measured by a cell-counting kit-8 (CCK8) assay. Data are expressed as a percentage of the control. (B) The oxygen consumption rate (OCR) and extracellular acidification rate (ECAR) were measured for HFD or LFD cells with a Seahorse XFe24 Flux Analyzer. The basal OCR, basal ECAR, and OCR/ECAR ratio are shown. (C) Absolute quantitative analysis of metabolites of the malate/aspartate shuttle in HFD or LFD cells. Data are presented as the mean ± SD. * p<0.05, ** p<0.01, *** p<0.001 as determined by an unpaired two-tailed Student's t-test.

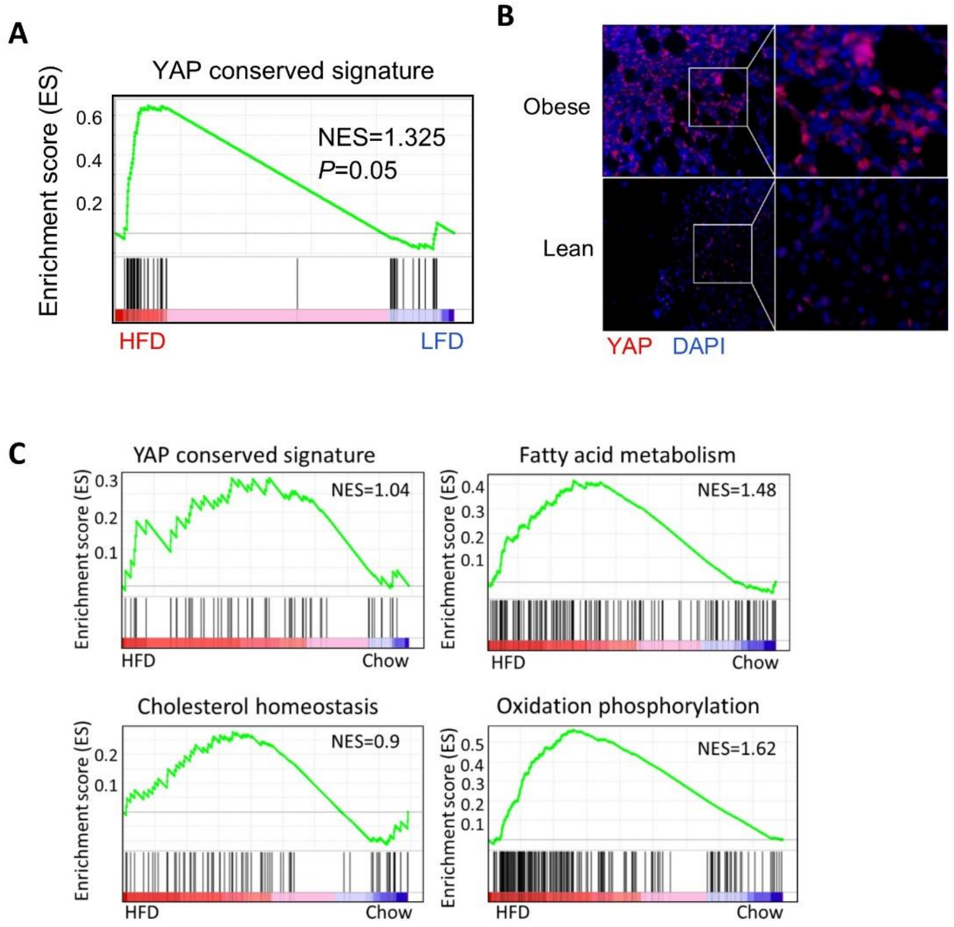

Supplementary Fig. 3 Obesity promotes YAP expression. (A) Gene set enrichment analysis (GSEA) of RNA-sequencing data for HFD or LFD cells. GSEA-enrichment plot of the YAP-conserved signature is shown. (B) Immunofluorescent images of YAP protein expressions in obese and lean mice tumor tissues. Enlarged pictures are shown in the right panel. (C) GSEA plots of the YAP, fatty acid metabolism, cholesterol homeostasis, and oxidative phosphorylation signatures in E0771 cells isolated from mice fed with HFD versus normal chow diet. Data were obtained from GSE165275 dataset.

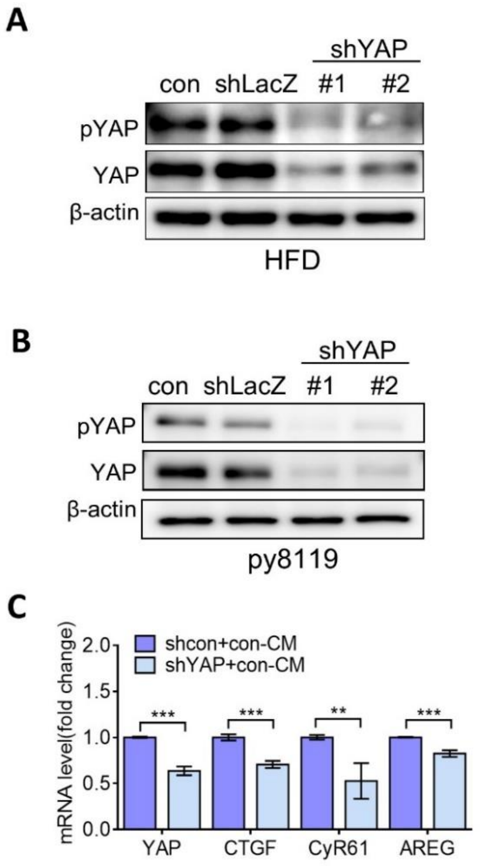

Supplementary Fig. 4 YAP knockdown efficiency in HFD and py8119 cells. (A) Western blot analysis of total and phosphorylated YAP levels in HFD, HFD/shLacZ, and HFD/shYAP cells. (B) Western blot analysis of total and phosphorylated YAP levels in Py8119, Py8119/shLacZ, and Py8119/shYAP cells. (C) qPCR analysis of YAP and YAP downstream gene (*CTGF*, *CyR61*, and *AREG*) expressions in Py8119/shLacZ, and Py8119/shYAP cells treated with control conditioned medium (con-CM). Data are presented as the mean ± SD. * $p<0.05$, ** $p<0.01$, *** $p<0.001$ as determined by an unpaired two-tailed Student's *t*-test.

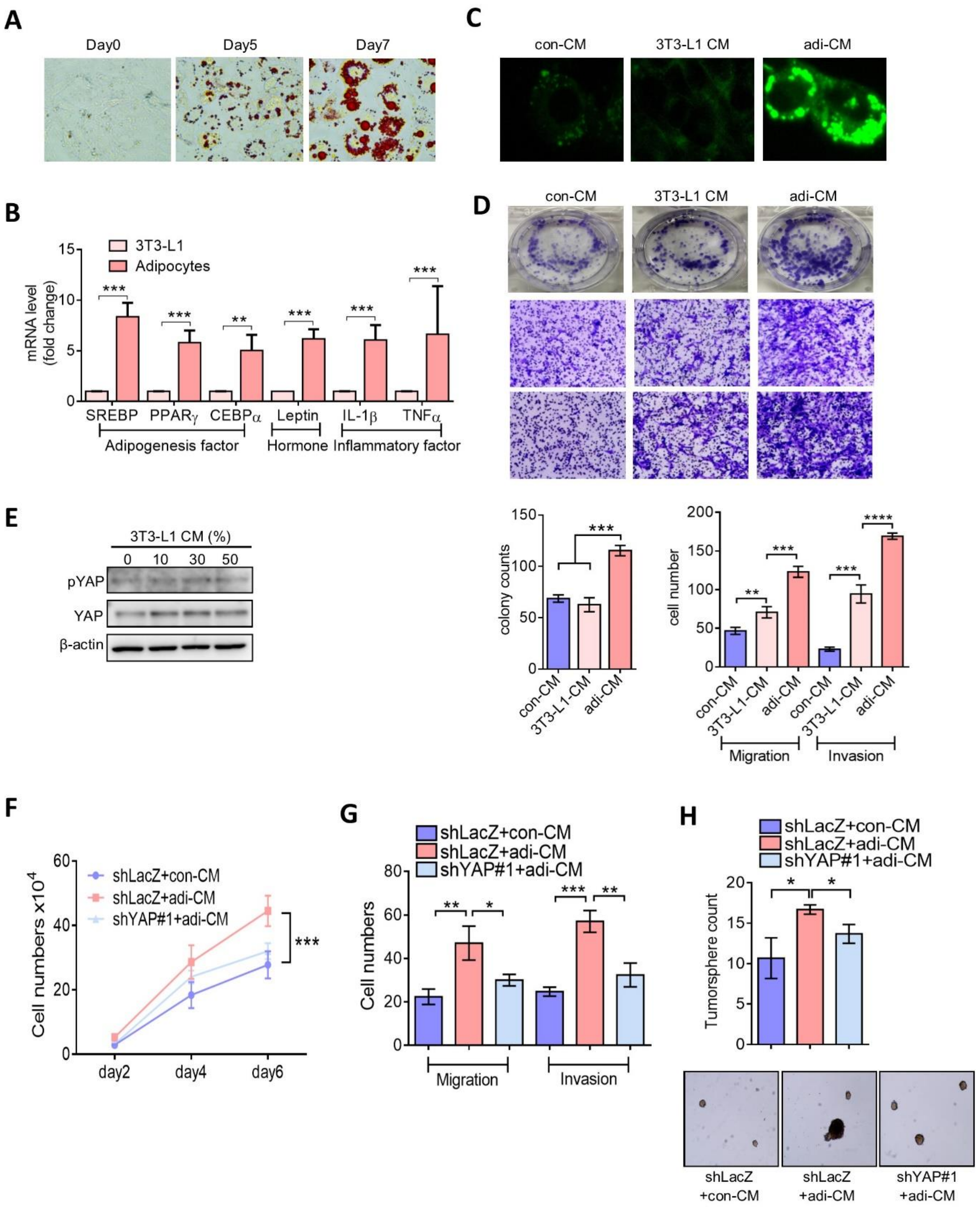

Supplementary Fig. 5 Adi-CM promotes aggressiveness of py8119 cells. (A) Oil red O staining during adipocyte differentiation from mouse 3T3-L1 cells. (B) qPCR analysis of adipocyte differentiation-associated genes in 3T3-L1 cells and differentiated adipocytes. (C) Lipi-green staining of Py8119 cells treated with con-CM, 3T3-L1-CM or adi-CM. (D) Colony formation assay and transwell analysis of py8119 cells treated with con-CM, 3T3-L1-CM or adi-CM.

Representative images of migration, invasion, and colony formation are presented (upper panel), and quantitative data (lower panel) are shown. (E) Western blot analysis of total and phosphorylated YAP levels in Py8119 cells treated with different concentrations of 3T3-L1-CM. (F) Cell proliferation of py8119, Py8119/shLacZ and Py8119/shYAP cells treated with adi-CM. (G) Transwell analysis of migration and invasion by py8119, Py8119/shLacZ, and Py8119/shYAP cells treated with adi-CM. Quantitative data are shown. (H) Tumorsphere formation assay of py8119, Py8119/shLacZ, and Py8119/shYAP cells treated with adi-CM. Representative images of tumorsphere formation are presented (left panel), and quantitative data (right panel) are shown. Data are presented as the mean ± SD. * $p<0.05$, ** $p<0.01$, *** $p<0.001$ as determined by an unpaired two-tailed Student's t-test.

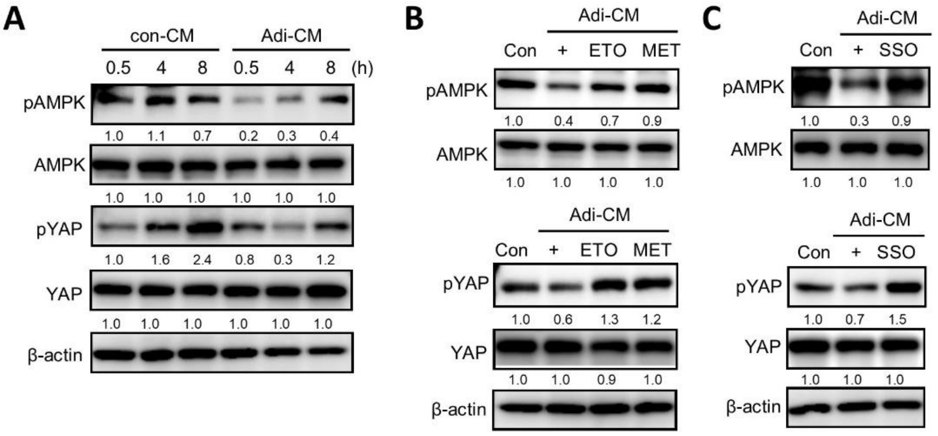

Supplementary Fig. 6 Adi-CM regulates the AMPK-YAP signaling in py8119 cells. (A) Western blot analysis of AMPK and YAP in py8119 cells treated with con-CM or adi-CM for different time intervals. (B) Py8119 cells were treated with adi-CM with or without inhibitors of FAO (etomoxir, ETO, 100 μM), AMPK (metformin, MET: 2 mM), and (C) fatty acid transporter (Sulfo-*N*-succinimidyl Oleate, SSO: 100 μM) for 8 h. Phosphorylation of AMPK and YAP were analyzed by Western blot. Quantification of protein level was carried out by using Image J software.

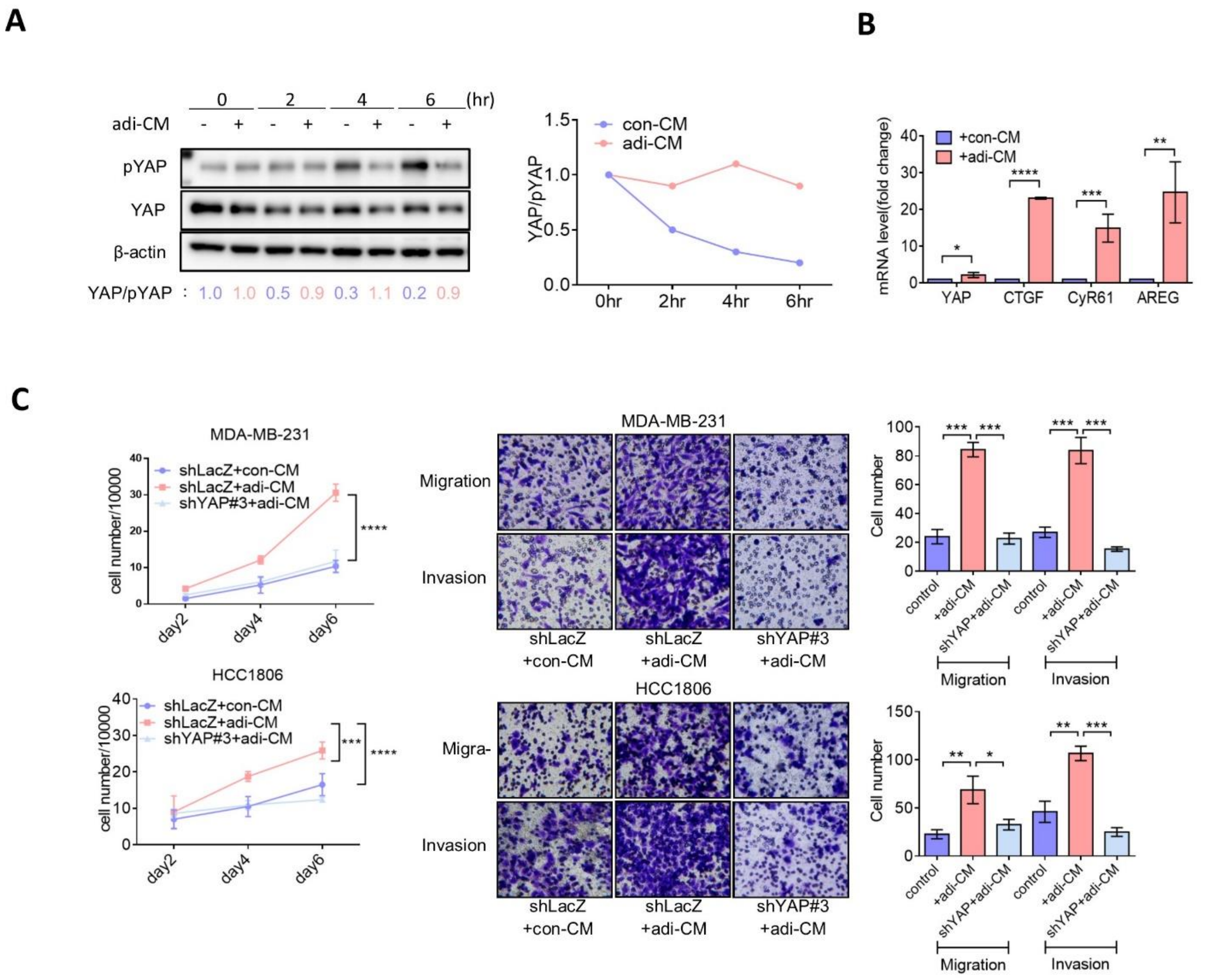

Supplementary Fig. 7 Adi-CM promotes aggressiveness in human TNBC cell lines, which requires YAP. (A) Western blot analysis of total and phosphorylated YAP levels in MDA-MB-231 cells treated with con-CM or adi-CM (left panel), and quantitative data (right panel) are shown. (B) qPCR analysis of YAP and YAP downstream gene (*CTGF*, *CyR61*, and *AREG*) expressions in Py8119 cells treated with con-CM or adi-CM. (C) MDA-MB-231, MDA-MB-231/shLacZ, and MDA-MB-231/shYAP cells were treated adi-CM for a cell proliferation assay and transwell analysis of migration and invasion. Data are presented as the mean ± SD. * p<0.05, ** p<0.01, *** p<0.001 as determined by an unpaired two-tailed Student's t-test.

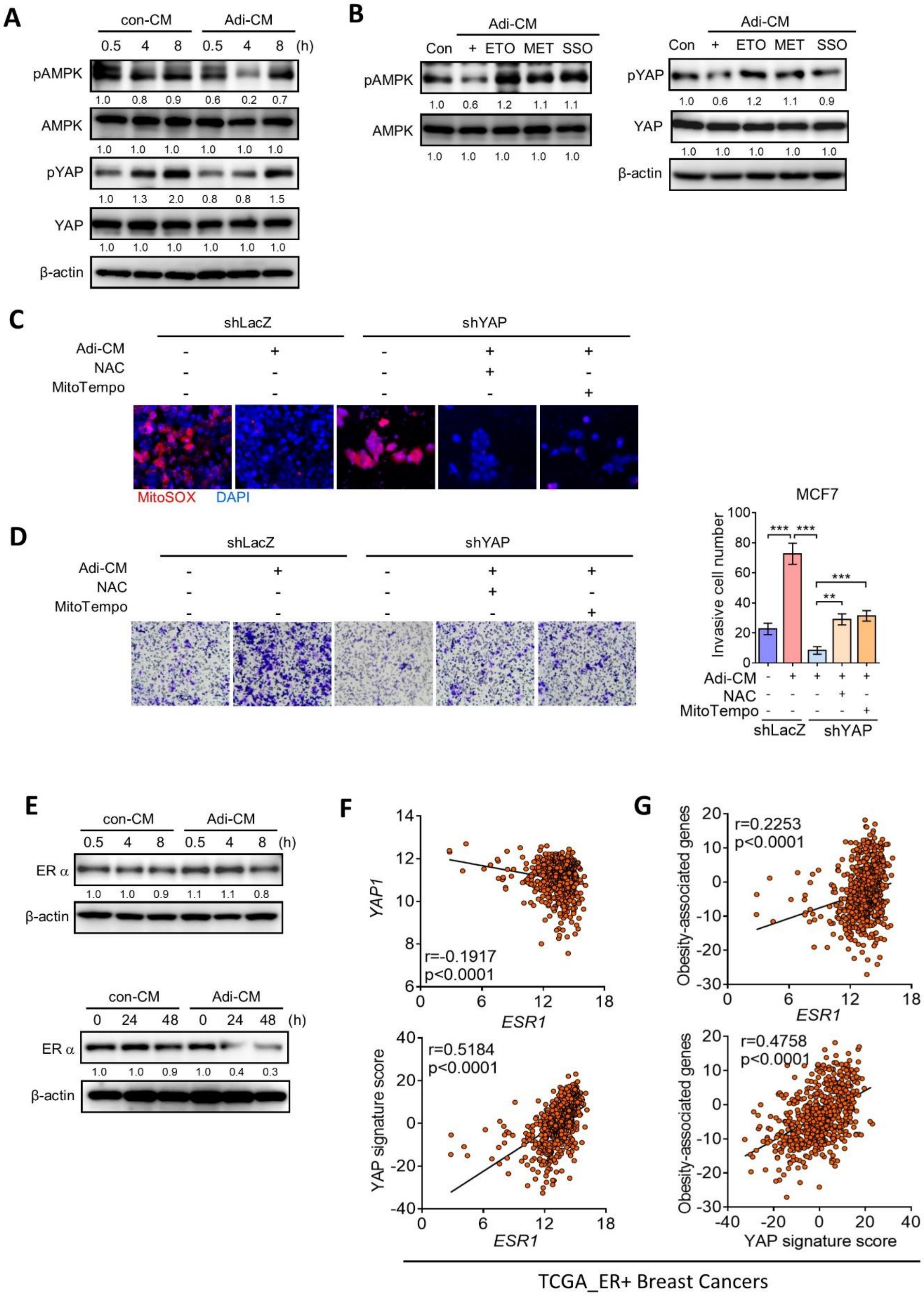

Supplementary Fig. 8 The effect of Adi-CM on ER+ MCF7 cells. (A) Western blot analysis of YAP and AMPK in MCF7 cells treated with con-CM or adi-CM for different time intervals. Quantification of protein level was carried out by using Image J software. (B) MCF7 cells were

treated with adi-CM with or without inhibitors of FAO (etomoxir, ETO, 100 μM), AMPK (metformin, MET: 2 mM), and fatty acid transporter (Sulfo-*N*-succinimidyl Oleate, SSO: 100 μM) for 8 h. Phosphorylation of YAP and AMPK were analyzed by Western blot. (C) Detections of mitochondrial ROS level and (D) cell migration in MCF7 cells in response to adi-CM and antioxidants. (E) Western blot analysis of ERα in MCF7 cells treated with con-CM or adi-CM for 0-8 h (upper panel) and 48 h (lower panel). Quantification of protein level was carried out by using Image J software. (F) Pearson correlations of *ESR1* with *YAP1* or YAP signature in ER+ breast cancers in TCGA database. (G) Pearson correlations of obesity-associated genes with *ESR1* or YAP signature in ER+ breast cancers in TCGA database.

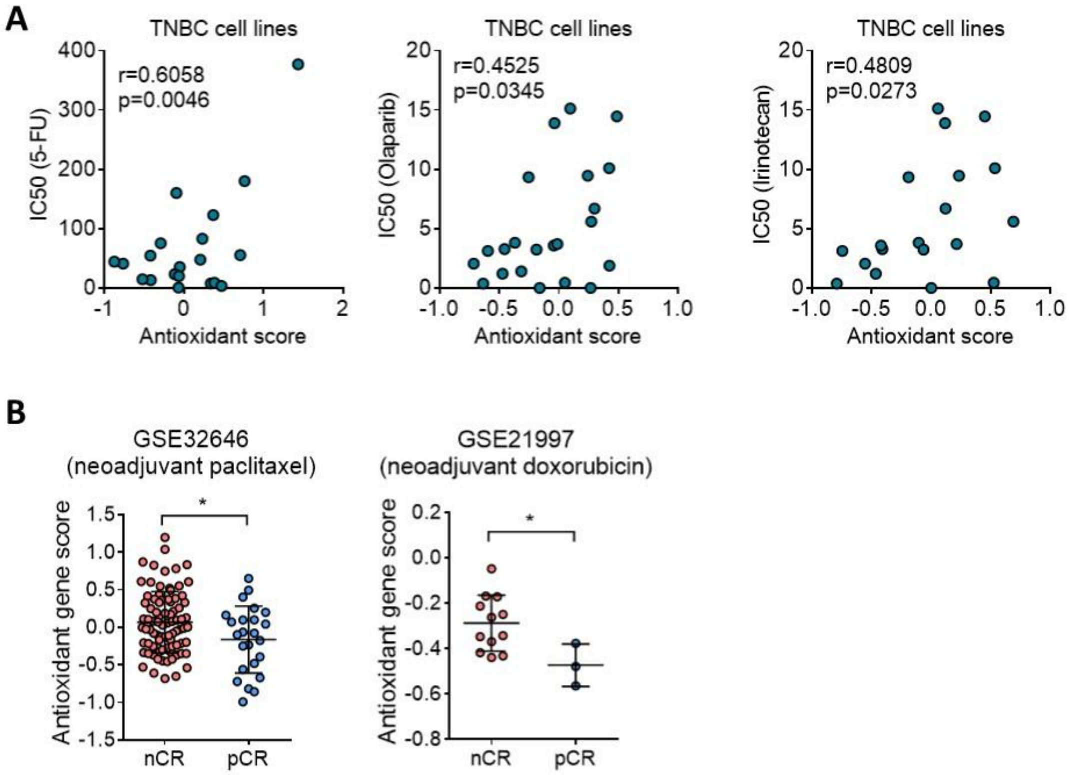

Supplementary Fig. 9 Obesity is associated with chemoresistance in TNBC. (A) Pearson correlation of the 50% inhibitory concentration (IC_{50}) values and antioxidant gene scores in TNBC cell lines. (B) Antioxidant gene scores in TNBC patients that were recorded to be pathologic complete response (pCR) or no complete response (nCR) after neoadjuvant doxorubicin or paclitaxel therapy. Data are presented as the mean ± SD. * $p<0.05$ as determined by an unpaired two-tailed Student's t-test.

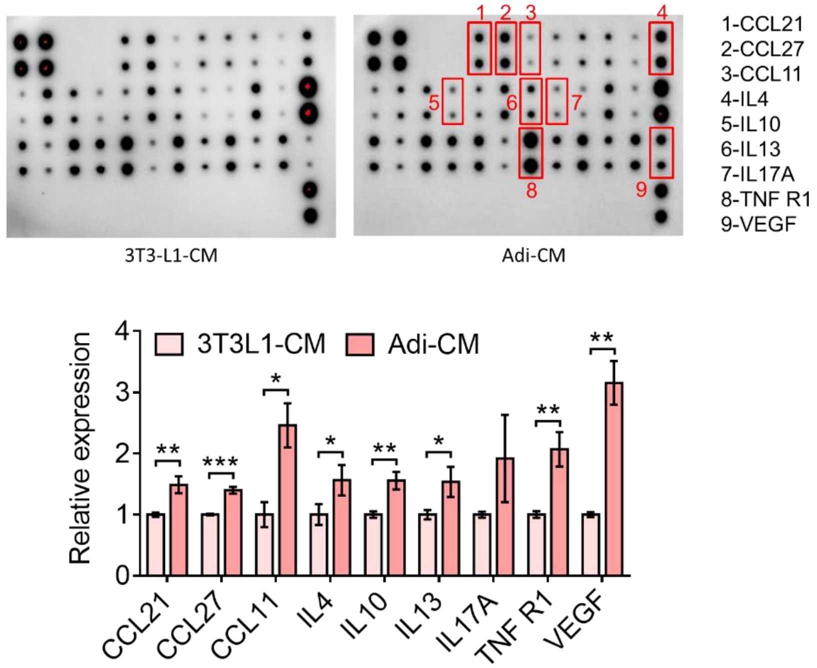

Supplementary Fig. 10 Cytokine array analysis of CM from adipocytes and 3T3L1. Densitometric analysis was carried out by using Image J software and data were normalized to the positive control, data were derived from two biological replicates and expressed as relative expression.

Supplemental Materials and Methods

Metabolite measurements

A lipid analysis was performed using mass spectrometry (MS). Cell pellets were extracted with 500 μL of an organic mixture composed of MeOH/MTBE/$CHCl_3$ (1.33:1:1, v/v/v). The extract was mixed for 1 h (900 rpm, at room temperature), and the supernatant was collected after centrifuging at 15,000 RCF for 10 min. The supernatant was evaporated to dryness with a centrifugal evaporator and was reconstituted with 50 μL of chloroform/methanol/water (60:30:4.5, v/v/v). The sample was further diluted with isopropyl alcohol (IPA)/acetonitrile (ACN)/H_2O (2:1:1, v/v/v) to 100 μL before a liquid chromatographic (LC)-MS analysis. The lipid extract was analyzed with a Waters Acquity UPLC system coupled to a Synapt G2 QTOF MS (Waters, Manchester, UK). An Acquity UPLC CSH column (100 × 2.1 mm, 1.7 μm) was used for lipid separation. The mobile phases were composed of 10 mM ammonium acetate and 0.1% formic acid in H_2O/ACN (40:60, v/v, solvent A), and 0.1% formic acid in ACN/isopropanol (10:90, v/v, solvent B). The gradient was set as: 0~0.5 min, 5% B; 0.5~1.5 min, 5%~32% B; 1.5~15.5 min, 32%~85% B; 15.5~15.6 min, 85%~97% B; 15.6~18 min, 97% B; 18~18.1 min; 97%~5% B; 18.1~20 min, 5% B. The flow rate was set to 0.3 mL/min, and the column temperature was maintained at 55 °C. Both positive and negative electrospray ionization modes were used to detect lipid components. The acquisition range of m/z was set from 100 to 2000 Da. The source temperature was set to 120 °C; the desolvation temperature was set to 450 °C; the cone voltage was set to 30 V for the positive mode and 25 V for the negative mode; and capillary voltages were set to 2800 (positive) and 2200 V (negative). A leucine-enkephalin solution (positive: m/z 556.2771, negative: m/z 554.2615) was used as the LockSpray internal reference. The collision energy used for the tandem MS (MS/MS) experiment was ramped from 13 to 45 V. Progenesis QI (vers. 2.4, Waters, Milford, MA, USA) was used for data processing in terms of peak alignment, normalization, signal integration, and initial compound assignments. Metabolite identification was

conducted by comparing the accurate masses, MS/MS fragment spectrum, and isotopic patterns to the METLIN mass spectral database and Human Metabolome Database (HMDB v4.0), and a LipidBlast. Heatmap was generated with MetaboAnalyst 4.0.

A targeted quantitative analysis of cellular metabolites was conducted with the C-SCOPE package of HMT (Human Metabolome Technologies) using capillary electrophoresis time-of-flight MS (CE-TOFMS) for the cation analysis and CE-MS/MS for the anion analysis. Briefly, 5×10^6 cells were washed twice with a 5% mannitol solution. Cells were treated with methanol and then treated with Milli-Q water containing internal standards (H3304-1002, HMT). The extract was filtered through a Millipore 5-kDa cutoff filter (UltrafreeMC-PLHCC, HMT) to remove macromolecules. The filtrate was centrifugally concentrated and resuspended in Milli-Q water for a metabolomic analysis by HMT. The metabolites were annotated based on the HMT metabolite database. A hierarchical cluster analysis (HCA) and principal component analysis (PCA) were performed with statistical analysis software (HMT). All values were normalized to cell numbers.

Cell viability

In total, 10^4 cells were seeded in 24-well plates and treated with different conditions as indicated. Cell viability was assessed by incubation with a Cell Counting Kit-8 (CCK8) reagent (Dojindo Molecular Technologies), and the absorbance of CCK8 was detected at a 450-nm wavelength with a spectrophotometer. Cell viability was expressed as the percentage of CCK8 reduction, with 100% assigned as the value of the absorbance of control cells.

RNA Isolation and Real-time Quantitative Polymerase Chain Reaction (qPCR)

Total RNA was extracted with a GENzolTM TriRNA Pure kit (Geneaid). Complementary (c)DNA was synthesized with M-MLV reverse transcriptase (Promega) and amplified with the GoTaq qPCR Master Mix (Promega) in a StepOne Plus Real-Time PCR system (Applied

Biosystems) with specific primers listed in Supplemental Table S1. Results were calculated using the ΔΔCT equation and are expressed as multiples of change relative to a control sample.

Western blotting

Total proteins were extracted with ice-cold RIPA buffer (50 mM Tris-HCl (pH 7.4), 150 mM NaCl, 1 mM EGTA, 1% Nonidet P-40, and 0.025% sodium deoxycholate) supplemented with protease and a phosphatase inhibitor cocktail (Roche Diagnostics). Equal amounts of protein were separated by sodium dodecylsulfate polyacrylamide gel electrophoresis (SDS-PAGE) and then transferred onto polyvinylidene difluoride membranes (Millipore). The membranes were blocked with 1% bovine serum albumin/TBST blocking buffer at room temperature for 30 min and incubated overnight at 4 °C with the primary antibody against YAP (#12395, Cell Signaling), phosphorylated (p)-YAP (S127) (#13008, Cell Signaling), and β-actin (GTX11003, GeneTex). The membrane was washed three times with TBST and further incubated with an appropriate horseradish peroxidase (HRP)-conjugated secondary antibody (Jackson ImmunoResearch) at room temperature for 1 h. Bands were visualized with an enhanced chemiluminescence system (Millipore) and detected with the BioSpectrum Imaging system (UVP). Quantification was carried out with Image J software.

Luciferase reporter assay

Promoter regions of MSRA (from position –900 to +100 bp), GCLC (from position–1651 to -1423 bp), PRDX1 (from position –1600 to +100 bp), and GSR (from position –1000 to +100bp) were amplified by GoTaq master mixes (Promega) and cloned into the pGL4.1[luc2] reporter plasmid. In total, 5×10^4 cells were seeded in 24-well plates and transiently transfected with the pGL4.1[luc2]-promoter plasmid together with the Renilla luciferase (RL)-TK plasmid using PolyJET transfection reagent for 24 h. Firefly and Renilla luciferase activities were

measured using a Dual-Luciferase Assay System (Promega) according to the manufacturer's instructions.

Chromatin immunoprecipitation (ChIP)

Protein-DNA complexes were crosslinked using 1% formaldehyde, which was then quenched by adding glycine. The chromatin complexes were extracted using a Chromatin Extraction Kit (ab117152) and sonicated to an average size of 250 bp with a MISONIX Sonicator 3000. The chromatin complexes were processed by a ChIP Kit Magnetic-One Step (ab156907) according to the manufacturer's protocol and incubated with anti-YAP (#14074, Cell Signaling) or mouse immunoglobulin G (IgG) overnight. The immunocomplexes were reverse-crosslinked, and purified DNA was subjected to a PCR analysis. The PCR primers are listed in Supplemental Table S1.

Flow cytometry and immunofluorescence

To analyze intracellular ROS, cells were treated with oleic acid (100 μM) for 0, 6, and 24 h and then incubated with 10 μM 2′,7′-dichlorofluorescein diacetate (DCFH-DA, Sigma Aldrich) for 30 min. For the mitochondrial ROS analysis, cells were incubated with 5 μM MitoSOX (ThermoFisher Scientific) for 15 min. Cells were trypsinized and washed twice with phosphate-buffered saline (PBS), and then analyzed with a BD FACSVia flow cytometer (BD Biosciences). For immunofluorescent staining, cells were seeded onto Millicell EZ slides (Millipore) for 24 h. Cells treated with oleic acid (100 μM) for 0, 6, and 24 h were incubated with 10 μM DCFH-DA for 30 min, 5 μM MitoSOX for 15 min, or 2 μM BODIPY 581/591 C11 (ThermoFisher Scientific) for 1 h. Cells cultured with adi-CM for 24 h were incubated with 0.5 μM Lipi-Green (Dojindo Molecular Technologies) for 30 min. Slides were fixed with 2% paraformaldehyde at room temperature for 10 min. Nuclei were counterstained with UltraCruz mounting medium (Santa Cruz Biotechnology).

Immunohistochemistry (IHC)

Formalin-fixed and paraffin-embedded tumor tissues were deparaffinized, rehydrated, and heated in an antigen-unmasking solution (Vector Labs). Slides were blocked with 3% BSA and incubated with anti-YAP (#12395, 1:250, Cell Signaling), anti-GSR (GTX114199, 1:200, Genetex), and anti-GCLC (ab53179, 1:200, Abcam) primary antibodies overnight at 4 °C and then incubated with SignalStain Boost IHC detection reagent (Cell Signaling Technology) at room temperature for 1 h. Slides were washed three times with TBST buffer and stained with 3,3'-diaminobenzidine (DAB) peroxidase substrate (Vector Laboratories).

Supplemental Table S1 List of oligonucleotides for the real-time PCR and ChIP

**Gene**	**Forward primer (5' to 3')**	**Reverse primer (5' to 3')**
m18sRNA	GTA ACC CGT TGA ACC CCA TT	CCA TCC AAT CGG TAG TAG CG
mMSRA	ATG AAG ATG GTT TGT GCC AAC A	CCA ATC CCC TGC ATG TGA A
mGSR	GAC ACC TCT TCC TTC GAC TAC C	CCC AGC TTG TGA CTC TCC AC
mPRDX1	AAT GCA AAA ATT GGG TAT CCT GC	CGT GGG ACA CAC AAA AGT AAA GT
mGCLC	GGG GTG ACG AGG TGG AGT A	GTT GGG GTT TGT CCT CTC CC
mYAP	CCC GAC TCC TTC TTC AAG C	CTC GAA CAT GCT GTG GAG TC
mCTGF	GCG CCT GTT CTA AGA CCT GT	TTC ATG ATC TCG CCA TCG GG
mCyR61	ATT CTT GAG TAG CAT TAG G	GTA CTA TGA AGC GAA GTC
mAREG	CTA TCT TTG TCT CTG CCA TCA	AGC CTC CTT CTT TCT TCT GTT
mSREBP	GTG AGC CTG ACA AGC AAT CA	GGT GCC TAC AGA GCA AGA G
mCEBPα	ACA CGG GAC TGA CGC AAC AC	AAC CCC GCA GGA ACA TCT TT
mPPARγ	GAA AGA CAA CGG ACA AAT CAC C	GGG GGT GAT ATG TTT GAA CTT G
mLeptin	TCC CTG CCT CAG ACC AGT G	TAG AGT GAG GCT TCC AGG ACG
mIL-1β	AAC CTG CTG GTG TGT GAC GTT C	CAG CAC GAG GCT TTT TTG TTG T
mTNFα	CAT CTT CTC AAA ATT CGA GTG ACA A	TGG GAG TAG ACA AGG TAC AAC CC
mGSR (-2146-41)	AGT ACT ATA AGG GAG ACA TG	TCT GTA ATG AGA CCT GAC AC
mGCLC (-1444-41)	TAG TGG GCA GTA AGT AGT G	ATC GAT GAA TGC ATG ATA GG
mPRDX1	ACC TGG TCC ATA GTA CTT AG	AGT CAA GGG ACA AAT GAA AG

(-1706-01)		
mMSRA *(-776-770)*	AAT TTC TGA AGT CCG TCT GG	ACA TCG ATC CAA AGT GAA AC
